# Structural insight of a concentration-dependent mechanism by which YdiV inhibits *Escherichia coli* flagellum biogenesis and motility

**DOI:** 10.1093/nar/gks869

**Published:** 2012-09-21

**Authors:** Bingqing Li, Ning Li, Feng Wang, Liming Guo, Yan Huang, Xiuhua Liu, Tiandi Wei, Deyu Zhu, Cuilan Liu, Hongfang Pan, Sujuan Xu, Hong-Wei Wang, Lichuan Gu

**Affiliations:** ^1^State Key Laboratory of Microbial Technology, Shandong University, Jinan 250100, ^2^College of Life Sciences, Hebei University, Baoding 071002 and ^3^Tsinghua-Peking Center for Life Sciences, Center for Structural Biology, School of Life Sciences, Tsinghua University, Beijing 100084, China

## Abstract

YdiV is a negative regulator of cell motility*.* It interacts with FlhD_4_C_2_ complex, a product of flagellar master operon, which works as the transcription activator of all other flagellar operons. Here, we report the crystal structures of YdiV and YdiV_2_–FlhD_2_ complex at 1.9 Å and 2.9 Å resolutions, respectively. Interestingly, YdiV formed multiple types of complexes with FlhD_4_C_2_. YdiV_1_–FlhD_4_C_2_ and YdiV_2_–FlhD_4_C_2_ still bound to DNA, while YdiV_3_–FlhD_4_C_2_ and YdiV_4_–FlhD_4_C_2_ did not. DNA bound FlhD_4_C_2_ through wrapping around the FlhC subunit rather than the FlhD subunit. Structural analysis showed that only two peripheral FlhD subunits were accessible for YdiV binding, forming the YdiV_2_–FlhD_4_C_2_ complex without affecting the integrity of ring-like structure. YdiV_2_–FlhD_2_ structure and the negative staining electron microscopy reconstruction of YdiV_4_–FlhD_4_C_2_ suggested that the third and fourth YdiV molecule bound to the FlhD_4_C_2_ complex through squeezing into the ring-like structure of FlhD_4_C_2_ between the two internal D subunits. Consequently, the ring-like structure opened up, and the complex lost DNA-binding ability. Thus, YdiV inhibits FlhD_4_C_2_ only at relatively high concentrations.

## INTRODUCTION

The formation and assembly of the flagellum, an essential motility apparatus in many bacteria, are well organized and hierarchically processed (1,2). At the top of the hierarchy is the *flhDC* operon, whose expression is required for the transcription of class II flagellar operons. The products of *flhDC* operon, FlhD and FlhC, form a heterohexamer (D_4_C_2_) to bind to the upstream of class II promoters and promote class II genes transcription (3). The DNA fragment bound by FlhD_4_C_2_, called the FlhD_4_C_2_ box, contains two 17- or 18-bp inverted repeats with a 10- or 11-bp spacer between them (4). It has been reported that the FlhC subunit interacts with DNA, while the FlhD subunit strengthens the specificity to the FlhD_4_C_2_ box and the stability of the protein–DNA complex (5,6). The two zinc-binding sites are located at both sides of the FlhDC heterohexamer complex, respectively. However, Wang *et al.* (7) have suggested that the DNA may mainly wrap around the FlhD subunits when FlhD_4_C_2_ activates the downstream gene transcription. The discrepancy is which subunit DNA wraps around.

The expression of flagellar genes is regulated on several levels by a series of regulators. The AMP-catabolite gene activator protein (CAP) complex activates *flhDC* transcription at high intracellular cAMP levels (8). Three other proteins RcsB, H-NS and RtsB regulate cell motility by interacting with the *flhDC* promoter. CsrA, an RNA-binding protein, up-regulates flagellar gene expression through enhancing translation of *flhDC* mRNA, while DnaK, a chaperone protein, converts native FlhDC into a functional transcriptional regulator. FlhD_4_C_2_ can be degraded by ClpXP protease (9–12). Recently, two other proteins, FliT and YdiV, are recognized as negative regulators that prevent FlhD_4_C_2_ from binding to its target DNA by direct protein–protein interactions (13,14). Interestingly, the two regulators exert their inhibitory roles in different modes, with FliT binding to the FlhC subunit and YdiV to the FlhD subunit. Although the crystal structure of FliT has been determined, it is still unclear how it shuts down the transcription through interaction with FlhD_4_C_2_ (15).

YdiV shares weak sequence similarity with typical EAL proteins. The EAL proteins, named by their characteristic motif ‘EAL’ (Glu-Ala-Leu), are often responsible for degradation of second signaling messenger bis-(3′-5′)-cyclic dimeric guanosine monophosphate (c-di-GMP) (16–18). The molecule regulates the switch between the motile and sessile lifestyles of bacteria: high intracellular concentrations of c-di-GMP promote biofilm formation, whereas low concentrations lead to motility. Therefore, the *in vivo* function of EAL proteins is predicted as stimulating bacterium motility by down-regulating the c-di-GMP level, like YhjH from *Escherichia coli* and STM1827 from *Salmonella *(19–21). Ten conserved residues of EAL proteins are essential for their catalytic activity and c-di-GMP binding (18,22,23).

YdiV does not show catalytic activity to c-di-GMP and cannot bind c-di-GMP (24,25). Eight out of the 10 conserved catalytic residues are not preserved in YdiV (23)*.* Interestingly, unlike typical EAL proteins, YdiV inhibits the cell motility rather than stimulating motility (26,27). Wada *et al.* (14) demonstrated that *Salmonella *YdiV functions as a novel anti-FlhD_4_C_2_ factor, which regulates bacterium motility and is responsible for nutritional control of the flagellum regulation in *Salmonella*. Under low-nutrient conditions, *Salmonella* is known to repress flagellum synthesis, while *E. coli* up-regulates flagellum synthesis (14,28). The different responses between *Salmonella *and *E. coli *raise the question whether YdiV in *E. coli* functions in the same way as its homologue from *Salmonella*, despite the fact that the sequence identity between them is 52%. Recently, Wada *et al.* (29) reported that *E. coli* YdiV can also inhibit motility and flagellum production by interacting with FlhD_4_C_2_, indicating that the inhibitory mechanisms of YdiV to FlhDC are similar between *E. coli* and *Salmonella*. Moreover, YdiV mediates the interaction between the two quorum sensing systems in *E. coli *in cooperation with its transcription activator SdiA and cAMP concentrations (30)*.* Recently, *Salmonella* YdiV has been highlighted to be required in the host–pathogen interactions, impacting *Salmonella* virulence by inhibiting flagellar genes in systemic tissues. YdiV mutant strain is vulnerable to caspase-1-mediated colonization restriction by lacking the function of fully repressed flagellin expression in systemic tissues (24,31). The regulation of virulence in *Salmonella* seems to be also caused by YdiV’s inhibition to flagellar biogenesis. However, the detailed molecular mechanism by which YdiV negatively regulates the transcriptional activity of FlhD_4_C_2_ remains unclear.

In this article, we report the crystal structures of YdiV at 1.9 Å resolution and the YdiV_2_–FlhD_2_ complex at 2.9 Å resolution. Our structure analyses combined with biochemistry studies and reconstruction of YdiV_4_–FlhD_4_C_2_ structure via negative staining electron microscopy provide a clear regulatory mechanism that stoichiometric binding of YdiV to FlhD in the FlhD_4_C_2_ complex results in the opening of the ring-like structure of FlhD_4_C_2_ with consequent loss of DNA binding. Very recently, Takaya *et al.* (32) have reported similar results that *Salmonella *YdiV forms multiple types of complexes with FlhD_4_C_2_ and the L22H substitution in FlhD prevents the interaction with YdiV. Our structure analysis gives us a clear explanation to those phenomena.

## MATERIALS AND METHODS

### Protein expression and purification

The ydiV and flhD genes were cloned into pET29b and pGL01 vector. YdiV was expressed in *E. coli* BL21(DE3) and purified by Ni^2+^-NTA affinity column, ion exchange column Source-Q and Superdex 200 successively. Se-Met-YdiV was expressed in *E. coli* BL21(DE3) using M9 medium. l-Seleno-methionnine was added to the culture when OD600 reached 0.5. The purification procedure of Se-Met-YdiV was the same with native YdiV. The YdiV–FlhD complex was obtained by co-expression in *E. coli* BL21 (DE3) with the same expression condition of YdiV. After Ni^2+^-NTA affinity column, the YdiV–FlhD complex was lysed by 0.25 mg/ml trypsin for 30 min and purified by ion exchange column Source-Q and Superdex 200 chromatography immediately. Whole flhDC operon was cloned into pET21b in which FlhC contained a C-terminal His-tag. The FlhD_4_C_2_ complex was obtained by co-expression.

### Site-directed mutagenesis

Mutants were constructed using the two-step PCR strategy and cloned into pGL01, respectively. Three YdiV mutants (A184E, F181A and F181A-A184E) were also cloned into pGEX-6P-1 and transformed into *Escherichia coli* str. K-12 substr. MG1655 for further motility studies.

### Crystallization and structure determination

Crystals were grown using hanging drop vapor diffusion at 20°C. Both native and anomalous diffraction data were collected at Shanghai Synchrotron Radiation facility (SSRF) beamline BL17u1. The data sets were processed using the HKL2000 software suite (33). Structure of YdiV alone was solved by single anomalous dispersion phasing. Five Se sites were found using the program SOLVE (34). Initial single anomalous dispersion phases were then improved and the chain was automatically traced using the program RESOLVE (35). The atomic model was built using COOT (36) and refined using PHENIX (37). The structure of YdiV–FlhD was determined at 2.9 Å resolution, with the molecular replacement approach using PHASER (38) with the FlhD structure (PDB code: 1G8E) and our YdiV structure as searching models. The model building and structure refinement of YdiV–FlhD follow the same procedure as YdiV structure. Data collection and structure refinement statistics are summarized in Supplementary Table S1. Structural figures were generated using PyMol (http://www.pymol.org).

### Size-exclusion chromatography

Two proteins (YdiV and FlhD_4_C_2_) were mixed in different mixing ratio (5:1, 1:1) for 10 min at room temperature and injected to size-exclusion chromatography using a Superdex 200 column. All data were processed by Origin.

### Protein pull-down assay

Bait proteins were prepared as described above. His-tag of prey proteins were removed by PPase during purification. Bait protein was immobilized onto Ni^2+^-NTA beads and then incubated prey protein at 4°C for 30 min. The mixture was washed three times using buffer containing 25 mM Tris–HCl (pH 8.0) and 100 mM NaCl. Proteins were eluted with elution buffer containing 25 mM Tris–HCl (pH 8.0), 100 mM NaCl and 250 mM imidazole. Then the elution samples were analyzed by SDS–PAGE with Coomassie blue staining. Prey proteins were incubated with Ni^2+^-NTA beads alone as a negative control.

### Electrophoretic mobility shift assay experiment

A 49-bp DNA fragment of the flhB promoter was synthesized as target DNA. Ten picomoles of DNA was pre-incubated with different ratios of proteins for 10 min in a reaction buffer containing 20 mM Tris–HCl (pH 8.0), 100 mM NaCl, 1 mM MgCl_2_, 1 mM ZnCl_2_ and 4%(v/v) glycerol. Then samples were analyzed using a native 5% polyacrylamide gel and dyed by ethidium bromide. Some gels were also dyed by Coomassie brilliant blue.

### Swarming motility assay

Motility was evaluated using 0.3% or 0.5% soft agar plates according to a reported method (26,27). Briefly, single colonies were poked into the plates using toothpicks and incubated for 6 h at 37°C, and then the diameter of motility was measured. For motility assays with the pGEX construct, colonies were picked from plates that contained 1 mM isopropyl β-d-1-thiogalactopyranoside. At least six independent colonies were checked for each strain.

### Negative staining EM sample preparation and single-particle image analysis

The gel filtration purified FlhDC–YdiV complex was diluted to 50–80 nM and immediately applied to glow-discharged holey carbon grids covered with a layer of thin carbon film. After 1 min, the samples were stained consecutively in three droplets of 2% (w/v) uranyl acetate solution for a total of 2 min, and the remaining stain was removed by gentle blotting with filter paper. The samples were examined using an FEI F20 electron microscope operated at 200 kV acceleration voltage using a nominal magnification of 50 000. Images were recorded on a 4 k × 4 k Ultrascan4000 CCD camera (Gatan) using low-dose mode with an exposure dose of ∼40 e/Å^2^. The defocus used to collect the raw image was −1.2 to −1.5 μm. The electron micrographs had a pixel size of 2.2 Å and were directly used for image processing. We used EMAN2 package to perform semi-automatic particle picking and to box the particles from the raw micrographs into boxes of 80 × 80 square pixels (39). The particles were normalized and high- and low-pass filtered prior all image processing procedures. About 30 000 raw particles of FlhDC–YdiV complexes were collected for two-dimensional reference-free alignment and classification using multivariate statistical analysis and multi-reference alignment in IMAGIC-4D (40) to a total of 200 classes.

## RESULTS

### Crystal structure of YdiV

**Figure 1.**
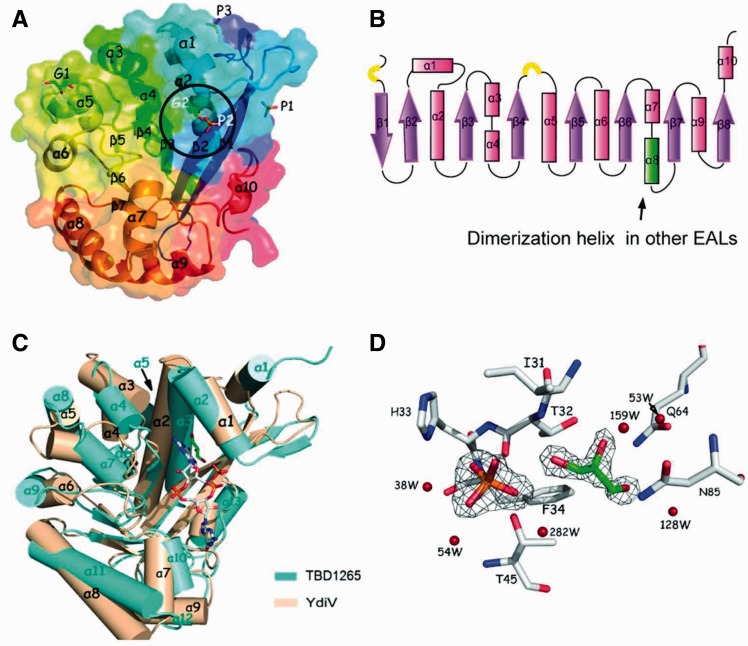
Crystal structure of YdiV. (**A**) Overview of YdiV. Structure of YdiV is shown in cartoon and surface mode using Mol A as a model with the secondary structure elements labeled. Phosphate and glycerol molecules in predicted c-di-GMP-binding site are marked in white color and enclosed by a black ellipse. (**B**) The topology view of YdiV structure. α-Helices are shown in pink and β-strands shown in purple. The 3_10_ helices are shown in yellow semicircular. Overall structure presents a TIM barrel-like fold. (**C**) Superposition of YdiV and its homologue TBD1265 (PDB code: 3N3T). The c-di-GMP legend is from structure of TBD1265, phosphate and glycerol exist in the same region in YdiV structure. (**D**) Interaction details and the electron density map of the phosphate and glycerol molecules in YdiV structure.

The YdiV structure was solved using selenium single-wavelength anomalous diffraction at 1.9 Å resolution. The final model of YdiV contains two protein molecules (Mol A and Mol B) per asymmetric unit. The YdiV monomer consists of 10 α-helices, 8 β-strands and 2 short 3_10_ helices, which exhibits a modified TIM-like barrel fold (Figure 1A and B). Although YdiV shares low-sequence identities with other EAL domain proteins (below 20% for the full length), the topology of the structures is the same. The lowest root mean square deviation of the superimposed Cα atoms between YdiV and its homologues is 3.0 Å (more than 223 Cα-positions of TBD1265, PDB code:3N3T). YdiV’s eight β-strands match well with those of TBD1265; in contrast, nearly all Cα atoms of the 10 α-helices show great spatial deviation. Particularly, the α8-helix of YdiV, which is essential for dimerization in other EAL structures, undergoes dramatic transformation (Figure 1C). Notably, although YdiV loses most of the residues coordinating with c-di-GMP, a similar groove that is responsible for c-di-GMP binding in other EAL structures is still retained. Interestingly, a phosphate and a glycerol molecule appear in this groove in contact with Thr32, His33, Phe34, Thr45, Gln64, Gln85 and some water molecules (Figure 1D). They are partially parallel to the c-di-GMP molecule in TBD1265, implicating that other small molecules with similar structures may bind to YdiV within this groove and play a regulatory role to the function of YdiV.

The interaction style of YdiV’s Mol A and Mol B is completely different from other dimerized EAL structures, such as Blrp1, YkuI, TBD1265, LapD and FimX (23,41–44). The Mol A and Mol B’s α8-helices, whose corresponding helices mediates the dimerization in Blrp1, YkuI, LapD and TBD1265 structures, are far away from each other (Supplementary Figure S1). The interaction interface between the two monomers is also smaller than that of the standard EAL protein dimers. We therefore speculate that YdiV does not form stable dimer in solution. This was confirmed by size-exclusion chromatography of purified YdiV (Supplementary Figure S2). The inability for YdiV to dimerize indicates its function in a unique way distinct from other EAL proteins.

### *E. coli* YdiV forms ternary complex with FlhDC through interacting with FlhD

YdiV is known to interact with FlhD_4_C_2_ in *Salmonella *(2011). To determine whether YdiV functions in the same way in *E. coli*, we co-expressed three groups of proteins: YdiV/FlhD-His, YdiV/FlhC-His and YdiV/FlhDC-His in *E. coli* BL21(DE3). YdiV formed stable complexes with both FlhD and FlhDC, but not with FlhC alone (Supplementary Figure S3A, B and C). These results indicate that YdiV binds to the FlhD_4_C_2_ complex through direct interaction with the FlhD subunit, and the binding of YdiV does not dissociate the FlhD and FlhC subunits in an FlhD_4_C_2_ complex. This is consistent with the report of Wada *et al.* Size-exclusion chromatography also confirmed that YdiV can form a stable ternary complex with FlhD_4_C_2_ (Supplementary Figure S3D). We further characterized the minimal YdiV-interacting domain in FlhD using purified recombinant proteins from *E. coli*. Four fragments of FlhD (1–71aa, 1–82aa, 1–98aa and 1–106aa) were tested with YdiV (Supplementary Figure S4). All fragments can form stable complex with YdiV in the nickel column pull-down assay, suggesting that the C-terminus of FlhD is not essential for YdiV binding, inconsistent with the *Salmonella* YdiV/FlhD interaction reported by Wade *et al.* (14).

### YdiV prevents FlhDC from binding to DNA in a quantity-dependent manner

**Figure 2.**
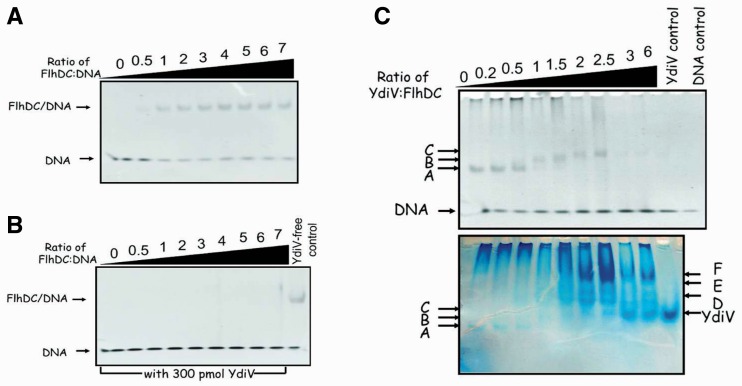
YdiV prevents FlhD_4_C_2_ from binding to DNA. (**A**) and (**B**) are parallel EMSA results without or with 5-fold excessive YdiV. Two reaction systems were almost the same, except that system B contains 300 pmol YdiV. The last lane of (B) was a positive control without YdiV. (**C**) EMSA results with different concentrations of YdiV. Sixty picomoles of FlhD_4_C_2_ was mixed with different ratio of YdiV for 10 min and then DNA-binding ability was examined by EMSA using 10 pmol DNA. The upper and lower pictures show the same gel dyed with ethidium bromide and Coomassie brilliant blue, respectively. All electrophoretic bands are named from A to F in order to distinguish. This is a representative image of two independent experiments.

To validate whether YdiV functions as an anti-FlhD_4_C_2_ factor and inhibits the DNA-binding activity of FlhD_4_C_2_ in *E. coli*, we performed a series of electrophoretic mobility shift assays (EMSAs) with a 49-bp promoter region of *flhB* that was proven to bind to FlhD_4_C_2_ (Figure 2A). We first added 5-fold excessive amount of purified YdiV protein into the DNA-binding system by FlhDC. As expected, no FlhDC–DNA complex was detected, indicating that YdiV does inhibit DNA binding by FlhD_4_C_2_ (Figure 2B). We then tested FlhDC’s DNA-binding behavior at different molar ratios of YdiV and FlhD_4_C_2_ (Figure 2C). Clear DNA shift can be detected when the molar ratio of YdiV:FlhD_4_C_2_ is below 2.5, whereas no shift happens upon the ratio exceeding 3. A surprising phenomenon was at the molar ratio of YdiV:FlhD_4_C_2_ below 2.5, the top DNA band shifted slower as the amount of YdiV increased, indicating the formation of larger complexes. More interestingly, when the gel was dyed with Coomassie brilliant blue, protein bands that could not be dyed with EB appeared at much slower positions in the gel. This unexpected phenomenon suggests that YdiV at different stoichiometry to FlhD_4_C_2_ leads to various effects on DNA binding of FlhD_4_C_2_. We thus speculate that YdiV can form a variety of complexes with FlhD_4_C_2_ (YdiV_1_–FlhD_4_C_2_, YdiV_2_–FlhD_4_C_2_, YdiV_3_–FlhD_4_C_2_ and YdiV_4_–FlhD_4_C_2_), in which YdiV_1_–FlhD_4_C_2_ and YdiV_2_–FlhD_4_C_2_ can still bind to DNA, while YdiV_3_–FlhD_4_C_2_ and YdiV_4_–FlhD_4_C_2_ lose DNA-binding ability. But this raises more questions such as why YdiV forms so many kinds of complexes with FlhDC and how it inhibits FlhD_4_C_2_ binding to DNA in a concentration dependent manner.

### Structure of YdiV–FlhD complex shows a heterotetramer

In order to elucidate the molecular mechanism by which YdiV regulates DNA-binding ability of FlhD_4_C_2_ complex, we embarked on structure determination of YdiV–FlhDC complex by x-ray crystallography. However, perhaps due to the structural heterogeneity of YdiV–FlhDC complex, numerous screening for the crystallization failed to give a hit. Despite this difficulty, we crystallized the YdiV–FlhD complex successfully. The structure was determined at 2.9 Å resolution with the molecular replacement approach using the FlhD structure (PDB code: 1G8E) and our YdiV structure as searching models. The final model contains four YdiV molecules (Mols A, C, E and G) and four FlhD molecules (Mols B, D, F and H) in the asymmetric unit, with each YdiV interacting with one corresponding FlhD in a uniform binding manner. Owing to limited proteolysis, some residues are missing. Two hundred and six residues (11–33 and 51–233) were built for YdiV and 81 residues (1–81) were built for FlhD.

**Figure 3.**
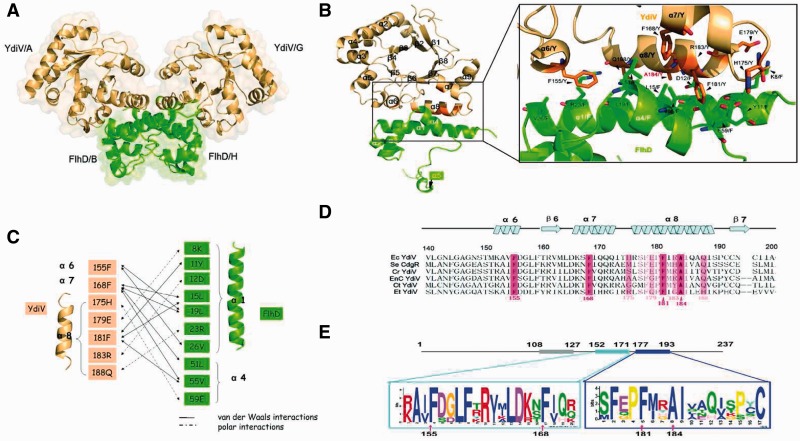
The YdiV–FlhD interaction. (**A**) Predicted tetramer structure of YdiV_2_–FlhD_2_ is shown in cartoon and surface mode using Mols A, B, G and H. YdiV and FlhD are highlighted in light orange and green, respectively. (**B**) YdiV–FlhD dimer and close-up view of interface are presented by Mols A and B. The α6, α7 and α8 of YdiV and the α1 and α4 of FlhD make up of this interface. Residues in the interface are shown in stick mode and labeled in relevant color (Y: from YdiV, F: from FlhD). (**C**) Schematic diagram denoting molecular interactions between YdiV and FlhD. Van der Waals interactions (<4 Å) and polar interactions (hydrogen bonds or salt bridges) between YdiV and FlhD are listed. (**D**) Sequence alignment of YdiV from different strains of *Enterobacteriaceae* group. Sixty residues (140–200 aa) of YdiV are used in this alignment and the most conserved residues are shown in pink. Ec: *Escherichia coli* str *K-12*, Se: *Salmonella enterica serovar*, Cr: *Citrobacter rodentium*, EnC: *Enterobacter cloacae*, Ct: *Cronobacter turicensis*, Et: *Erwinia tasmaniensis. *(**E**) Conserved motifs predicted by MEME suite. Three conserved motifs with ∼20 residues were found out and two of them (152–171 aa and 177–193 aa) located in the region of interface with FlhD. The most conserved residues of YdiV are marked by arrowhead.

The interaction interface analysis using CCP4 showed that the YdiV–FlhD complex may form a stable tetramer (Mol ABHG or Mol CDFE) in solution through a tightly coupled FlhD dimer core (Mol BH or Mol DF) (Figure 3A). The dimerization pattern of FlhD in the YdiV–FlhD complex is very similar to that of FlhD homodimers (PDB code:1G8E), where residues Cys 65/B and Cys 65/H are connected by a disulfide bond (45). The size-exclusion chromatography results also showed that YdiV–FlhD complex exists as tetramer in solution (Supplementary Figure S5). Figure 3B illustrates details of the YdiV–FlhD interface using Mol A and Mol B as example. The interface spans 973 Å^2^, which accounts for nearly 10% of the total YdiV surface area and 15% of FlhD. It consists of α6, α7 and α8 of YdiV and α1 and α4 of FlhD which constitutes hydrogen bonds, salt bridges and hydrophobic interactions. Residues Phe155, Phe168, His175, Glu179, Phe181, Arg183 and Gln188 of YdiV and Lys8, Tyr11, Asp12, Leu15, Leu19, Arg23, Val26, Leu51, Val55 and Glu59 of FlhD directly participate in the interaction and compose a highly firm interface (Figure 3C). Remarkably, α8 (176–192) of YdiV parallelizes and intensively interacts with α4 (5–26) of FlhD, providing most of the interacting residues. Side chains of three aromatic residues (Phe155, Phe168 and Phe181) from YdiV anchor onto α1 and α4 in the adjacent hydrophobic pockets of FlhD and form a hydrophobic core at the center of the molecular interface. The interface is further secured by a network of polar contacts: His175^Y^/Glu59^F^, Glu179^Y^/Lys8^F^ and Arg183^Y^/Aap12^F^. Compared with their single structures, YdiV and FlhD do not show significant conformational change even in the interface region. However, the side chains of the essential residues (His175, Glu179, Phe181 and Arg183) of YdiV, which mediate the interactions, show obvious deflection (Supplementary Figure S6). Very recently, Takaya *et al.* reported that the L22H substitution in FlhD prevents its interaction with YdiV. Leu22 of *Salmonella *FlhD, which is Leu19 of *E. coli* FlhD in our structure, is located in the interface of YdiV–FlhD. The side chain of His is hydrophilic and larger than Leu, so it may interrupt the interaction between these two proteins (32).

Moreover, sequence alignment shows that the key residues (Phe155, Phe168, Phe181 and Ala184) of YdiV involved in the interface are highly conserved across *E. coli* and other *Enterobacteriaceae* group orthologs (Figure 3D and E). As we input six sequences including YdiV and its orthologs into the motif prediction tool MEME suite, three conserved motifs with about 20 residues were detected and two of them were located within the interface. The high conservation of residues composing the interface of YdiV and FlhD across *Enterobacteriaceae* group suggests that YdiV from other *Enterobacteriaceae* group members may also interact with FlhD in the same way and down-regulate flagella biogenesis and motility.

### YdiV can squeeze into the ring-like structure of FlhD_4_C_2_ complex

**Figure 4.**
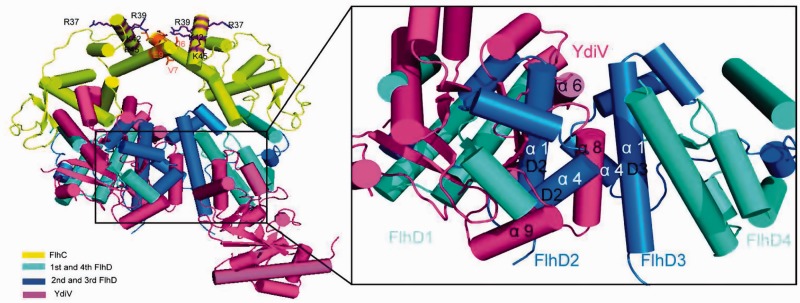
Superposition of YdiV–FlhD structure and FlhD_4_C_2_ structure. The structure of FlhD_4_C_2_ is used as a model, and YdiV–FlhD is superimposed to the third and the fourth FlhD molecules of FlhD_4_C_2_, respectively (YdiV: hotpink, FlhC: yellow, FlhD: blue). Whole structures are shown in cartoon mode and residues of FlhC involved in mutation studies are shown in sticks. The right figure presents a close-up view of interface between the second and the third FlhD molecules of FlhD_4_C_2_. The α1 and α4 of the third FlhD mediate both the YdiV–FlhD interaction and the second–third FlhD interaction. Two interfaces are overlapped, and the interface of YdiV–FlhD seems more solid than D2–D3. Helices from FlhD are marked in white color and the ones from YdiV in black.

**Figure 5.**
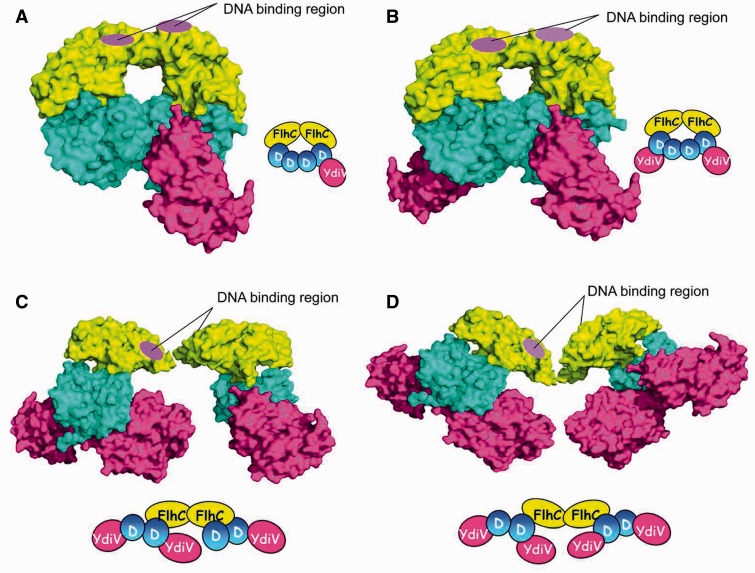
Interaction models between various numbers of YdiV and FlhD_4_C_2_. (**A**) One YdiV molecule binds to the fourth FlhD molecule of FlhD_4_C_2_. The adding of YdiV is done by superposition of YdiV–FlhD structure and FlhD_4_C_2_ structure (YdiV: hotpink, FlhC: yellow, FlhD: blue). The positive-charge-enriched FlhC regions that are necessary for DNA binding are highlighted by purple circle. (**B**) Two YdiV molecules are added to the first and fourth FlhD molecules of FlhD_4_C_2_. The six-membered ring structure of FlhD_4_C_2_ remains intact. (**C**) Three YdiV molecules are added to the first, second and fourth FlhD molecules of FlhD_4_C_2_. The six-membered ring conformation of FlhD_4_C_2_ is destroyed. The relative position of FlhC monomers in FlhC dimer changed as a result of the steric hindrance. Two DNA-binding regions locate at both the front and back sides, and only the front one is highlighted. (**D**) Four YdiV molecules bind to the FlhD of FlhD_4_C_2_. The relative positions of the FlhC subunits show a significant change.

YdiV forms stable heterotetramer with FlhD and can form quaternary complex with FlhD_4_C_2_ through interacting with FlhD. Why can it form a variety of complex with FlhD_4_C_2_ in a concentration-dependent manner? In order to explain this phenomenon, we superimposed the FlhD subunit in the YdiV–FlhD model onto the four FlhD subunit in FlhD_4_C_2_ model, respectively (Figure 4). Very surprisingly, the four FlhD subunits of FlhD_4_C_2_ adopt different structures. The α1 and α4 of the two peripheral FlhD subunits which compose the main part of the binding interface with YdiV are exposed. These two α-helices of the two internal FlhD subunits are completely buried in the central interface. In this means, only the two peripheral FlhD subunits of FlhD_4_C_2_ are accessible for YdiV binding without affecting the integrity of FlhD_4_C_2_ complex. So we speculate at low stoichiometry, YdiV binds sequentially to the two peripheral FlhD subunits to form two kinds of quaternary complexes (YdiV_1_–FlhD_4_C_2_ or YdiV_2_–FlhD_4_C_2_ shown in Figure 5A and B). With the increase of the ratio of YdiV, the exposed sites of FlhD for YdiV to bind are saturated. Hereafter, YdiV has to squeeze into the ring-like structure of FlhD_4_C_2_ in order to bind to the internal FlhD subunits, which destroys the interface between the central FlhD subunits and leads to significant structural reorganization of the protein complex (Figure 5C and D). At this stage, two kinds of quaternary complexes (YdiV_3_–FlhD_4_C_2_ or YdiV_4_–FlhD_4_C_2_) could form. As the ratio of YdiV keeps increasing, the internal binding sites of FlhD for YdiV are eventually saturated and the final product becomes YdiV_4_–FlhD_4_C_2_.

**Figure 6.**
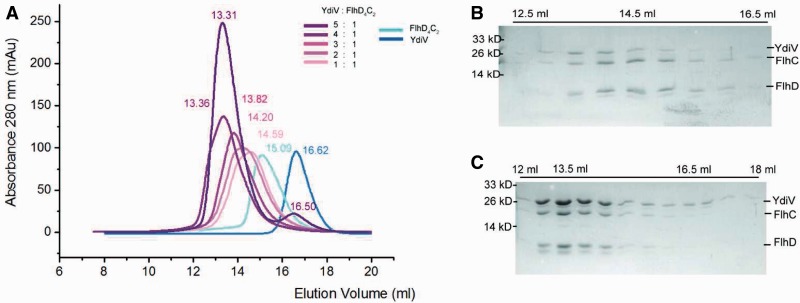
A stable hetero-decameric YdiV_4_–FlhD_4_C_2_ complex exists in solution. (**A**) Size-exclusion chromatography results of 5:1, 4:1, 3:1, 2:1 or 1:1 mixture of YdiV and FlhD_4_C_2_. Single YdiV and FlhD_4_C_2_ as controls. The elution volume of every peak value is marked in corresponding colors. (**B**) SDS–PAGE results of the elutions of 1:1mixture from 12.5 to 16.5 ml. The lane with a peak value of 14.5 ml is highlighted. Peak value lane is highlighted by 14.5 ml. (**C**) SDS–PAGE results of the elutions of 5:1 mixture from 12 to 18 ml. The lanes of two peak values at 13.5 and 16.5 ml are highlighted.

When the FlhD_4_C_2_ complex is saturated by YdiV, two YdiV molecules will squeeze into the ring-like structure. Severe steric hindrance has to be overcome during this process (Figure 5D). The associated significant structural reorganization of the protein complex raises a question whether the YdiV_4_–FlhD_4_C_2_ complex exists stably in solution. In order to answer this question, we performed size-exclusion chromatography (Figure 6). YdiV and FlhD_4_C_2_ were mixed at molar ratios of 5:1, 4:1, 3:1, 2:1 or 1:1 and loaded onto a size-exclusion column. Single YdiV or FlhD_4_C_2_ was examined in the same condition as control. The YdiV–FlhD_4_C_2_ mixture with the molar ratio of 1:1 had a single elution peak at a position (14.59 ml) larger than FlhD_4_C_2_ hexamer (15.09 ml) (SDS–PAGE result of elution solution shown in Figure 6B). As the YdiV proportion increased, the elution peak of the mixtures came out earlier from the column until reached at 13.31 ml. The 5:1 YdiV–FlhD_4_C_2_ mixture showed two peaks (13.31 and 16.50 ml) indicating the presence of excessive amount of free YdiV in the system. Figure 6C showed gel result of elution samples of 5:1 YdiV–FlhD_4_C_2_ mixture: the first peak was FlhD_4_C_2_Y_4_ complex; the second one was excessive YdiV. Mixtures of YdiV and FlhD_4_C_2_ at different proportions were also analyzed using Source-Q column (Supplementary Figure S7). The YdiV_4_–FlhD_4_C_2_ complex was eluted as a single peak at the conductance around 16.1 mS/cm. All the observations indicated that the binding of four YdiV molecules does not lead to the dissociation of FlhD_4_C_2_, and heterodecameric YdiV_4_–FlhD_4_C_2_ complex exists stably in solution.

### DNA binds the FlhD_4_C_2_ complex through wrapping around FlhC subunits and the YdiV_4_–FlhD_4_C_2_ complex loses ring-like structure and DNA-binding ability

**Figure 7.**
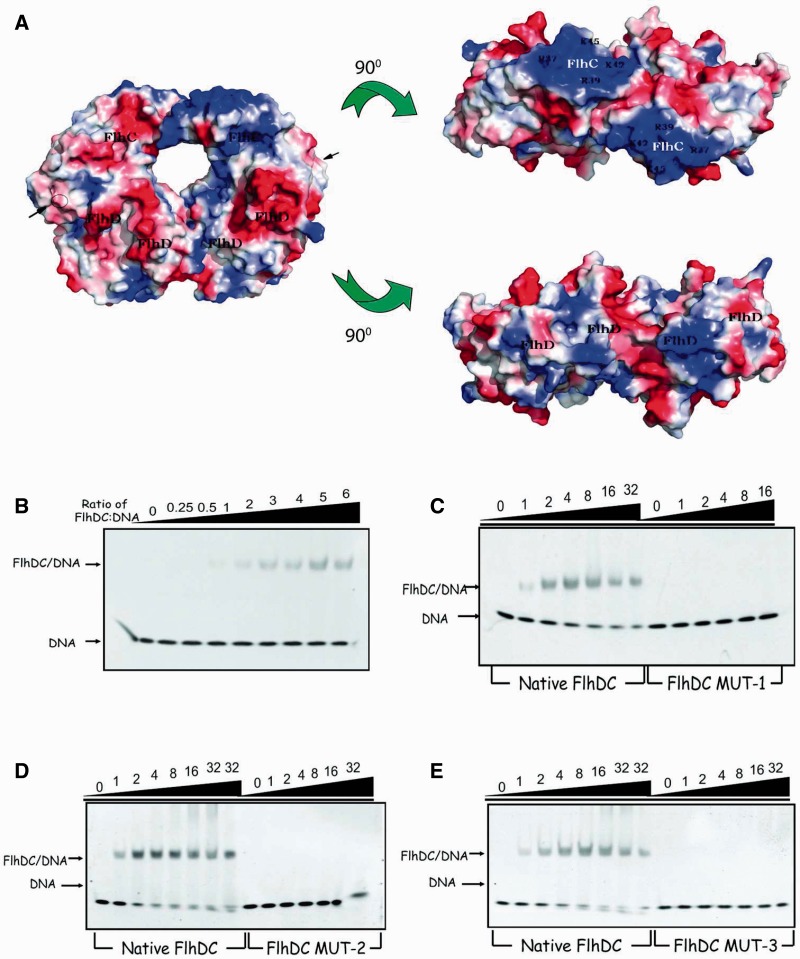
The DNA-binding affinity of FlhD_4_C_2_ and three mutants. (**A**) Vacuum electrostatics view of FlhD_4_C_2_. The structure of FlhD_4_C_2_ (PDB code: 2AVU) is shown in surface mode with vacuum electrostatics (red, negative; blue, positive). Three views related by two 90° rotations. Two FlhC subunits are marked in white color, and basic residues using in mutation and FlhDs are marked in black. (**B**) EMSA results of native FlhD_4_C_2_ and *flhB* promoter DNA. Ten picomoles of DNA was pre-incubated for 10 min with different ratios FlhD_4_C_2_ and run in a native 5% polyacrylamide gel at 4°C and then dyed by ethidium bromide. The ratio of protein to DNA is from 0 to 6. (**C**) Comparison of the DNA-binding affinity of native FlhD_4_C_2_ and DCMut-1. The front seven lanes were positive control and the others were done by the same reaction system and same method, except that native FlhD_4_C_2_ protein was replaced by DCMut-1 protein. The ratio of protein to DNA is from 0 to 32. (**D**) EMSA comparison of native FlhD_4_C_2_ and DCMut-2. (**E**) EMSA comparison of native FlhD_4_C_2_ and DCMut-3.

Our results show that YdiV can form a variety of complex with FlhD_4_C_2_. Then why can YdiV_1_–FlhD_4_C_2_ and YdiV_2_–FlhD_4_C_2_ still bind to DNA, whereas YdiV_3_–FlhD_4_C_2_ and YdiV_4_–FlhD_4_C_2_ lose DNA-binding ability? The crystal structure of the FlhD_4_C_2_ shows two zinc-binding sites located close to the interface of FlhD and FlhC. However, the DNA-binding manner remains unclear (8). If DNA wraps around FlhD subunits, YdiV should displace DNA from the FlhD_4_C_2_ complex when YdiV_1_–FlhD_4_C_2_ or YdiV_2_–FlhD_4_C_2_ complex are formed. Why is the DNA fragment released only when more YdiV molecules incorporate into the complex? Vacuum electrostatic analysis of FlhD_4_C_2_ displays a large positively charged region on each FlhC subunit (Figure 7A). The positively charged region on FlhD subunit is much smaller than on FlhC subunit indicating that FlhC may be more competitive than FlhD in binding to the negatively charged DNA. Thus, we designed three mutants (DCmut-1, DCmut-2 and DCmut-3) of FlhC to verify the DNA-binding mechanism. Lys42 and Lys45 of FlhC were substituted by Glu in DCmut-1; DCmut-2 contains four substitutions (R37E, R39E, K42E and K45E). DCmut-3 (I6D, V7D and E7A) was designed to destroy the dimerization interface of FlhC in FlhD_4_C_2_. During purification, the FlhDC mutants behave similarly to the wild-type FlhDC complex (Supplementary Figure S8). DCmut-1 and DCmut-2 possess similar secondary structure with native FlhDC complex while DCmut-3 presents a little difference when determined by circular dichroism spectroscopy. Pull-down assays showed that all FlhDC mutants can still interact with YdiV. The elution volume of DCmut-3 was larger than the wild-type FlhDC complex and other two mutants, indicating that DCmut-3 fails to dimerize in solution.

EMSA was used to detect the DNA–protein interaction with the 49-bp promoter region of flhB used as the target DNA. When the ratio of wild-type FlhDC and DNA exceeds 1:1, FlhDC-DNA complex can be detected (Figure 7B). Compared with the wild-type FlhDC protein, DCmut-1 and DCmut-2, whose basic residues (R37, R39, K42 and K45) were substituted by polar acidic ones, cannot bind DNA anymore even with 16-fold excess (Figure 7C and D). DCmut-3 also entirely abolished FlhDC’s DNA-binding ability (Figure 7E).

Previous studies showed that FlhD did not bind to DNA, while FlhC bound to DNA independently but with a binding affinity less than one-tenth of FlhD_4_C_2_ (6,7). Both DCmut-1 and DCmut-2 lost the ability of DNA binding, indicating that the selected amino acid residues on FlhC play an irreplaceable role in DNA binding. Furthermore, DCmut-3, which was constructed to break the interface of FlhC dimer and to destroy the ring-like structure of FlhD_4_C_2_, also did not bind to DNA even in a 32-fold excess. Our data strongly suggest that target DNA directly interacts with the positive-charge-enriched region of FlhC, while FlhD is essential for DNA binding by keeping the ring-like structure of FlhD_4_C_2_.

Given the above-mentioned data, we can explain how YdiV regulates DNA binding of FlhD_4_C_2_ in a concentration-dependent manner. The FlhD_4_C_2_ complex recruits DNA through the Zn-Cys cluster and the positive-charge-enriched region of FlhC dimer. In this process, the ring-like structure of FlhD_4_C_2_ is indispensable. Through the interaction with FlhD, YdiV can form a variety of complexes with FlhD_4_C_2_. At low stoichiometric concentration, YdiV only binds to the offside of FlhD_4_C_2_ and do not affect DNA-binding affinity of FlhD_4_C_2_. At high stoichiometric concentration, the third and fourth YdiV molecules squeeze into the ring-like structure of FlhD_4_C_2_ and induce structure rearrangement. So the DNA-binding site is no longer suitable for DNA binding.

**Figure 8.**
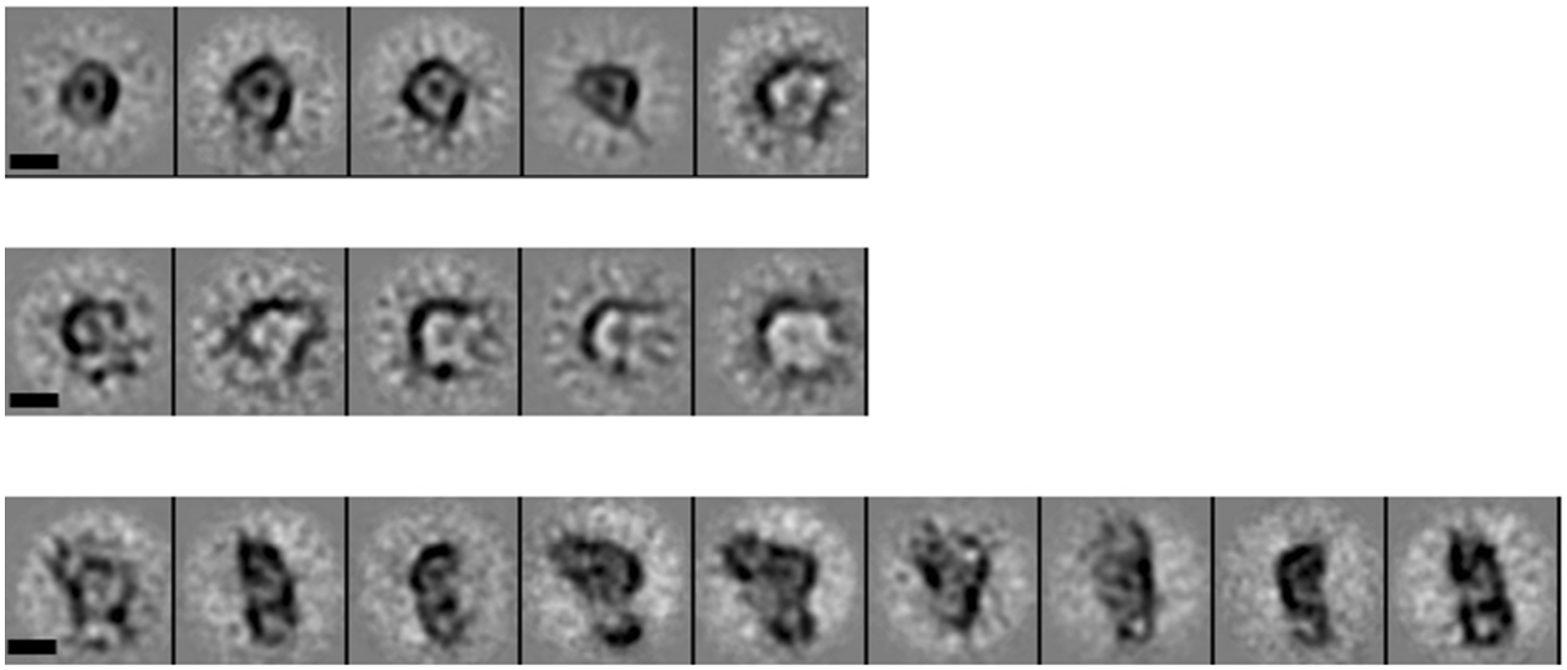
Two-dimensional single-particle EM class averages of FlhDC and FlhDC-YdiV complexes showing various conformations. The top row is ring-shaped class averages representing apo–FlhDC complexes. The middle row is half-opened FlhDC complexes bound with YdiV molecules. The bottom row is wide opened FlhDC complexes bound with YdiV molecules. The scale bars represent 5 nm.

To confirm that the YdiV_4_–FlhD_4_C_2_ complex lost ring-like structure, we performed negative staining EM of YdiV_4_–FlhD_4_C_2_ complex. Indeed, two-dimensional class averages of YdiV_4_–FlhD_4_C_2_ complexes showed various conformations, among which both half-opened and wide-opened YdiV_4_–FlhD_4_C_2_ complexes were observed (Figure 8). As a control, FlhD_4_C_2_ without YdiV showed integral ring-like structure.

### YdiV mutants do not bind to FlhD, affect the DNA binding of FlhD_4_C_2_ or inhibit cell motility *in vivo*

Mutagenesis was employed to validate the results of structure analysis. Eight residues (Phe155, Phe168, His175, Glu179, Phe181, Arg183, Ala184 and Gln188) of YdiV are in contact with FlhD. Most of the interacting residues are located on α8 (176–192aa), which parallelizes and intensively interacts with α4 (5–26aa) of FlhD. We constructed 12 mutants for YdiV and investigated their binding abilities with FlhD.

**Figure 9.**
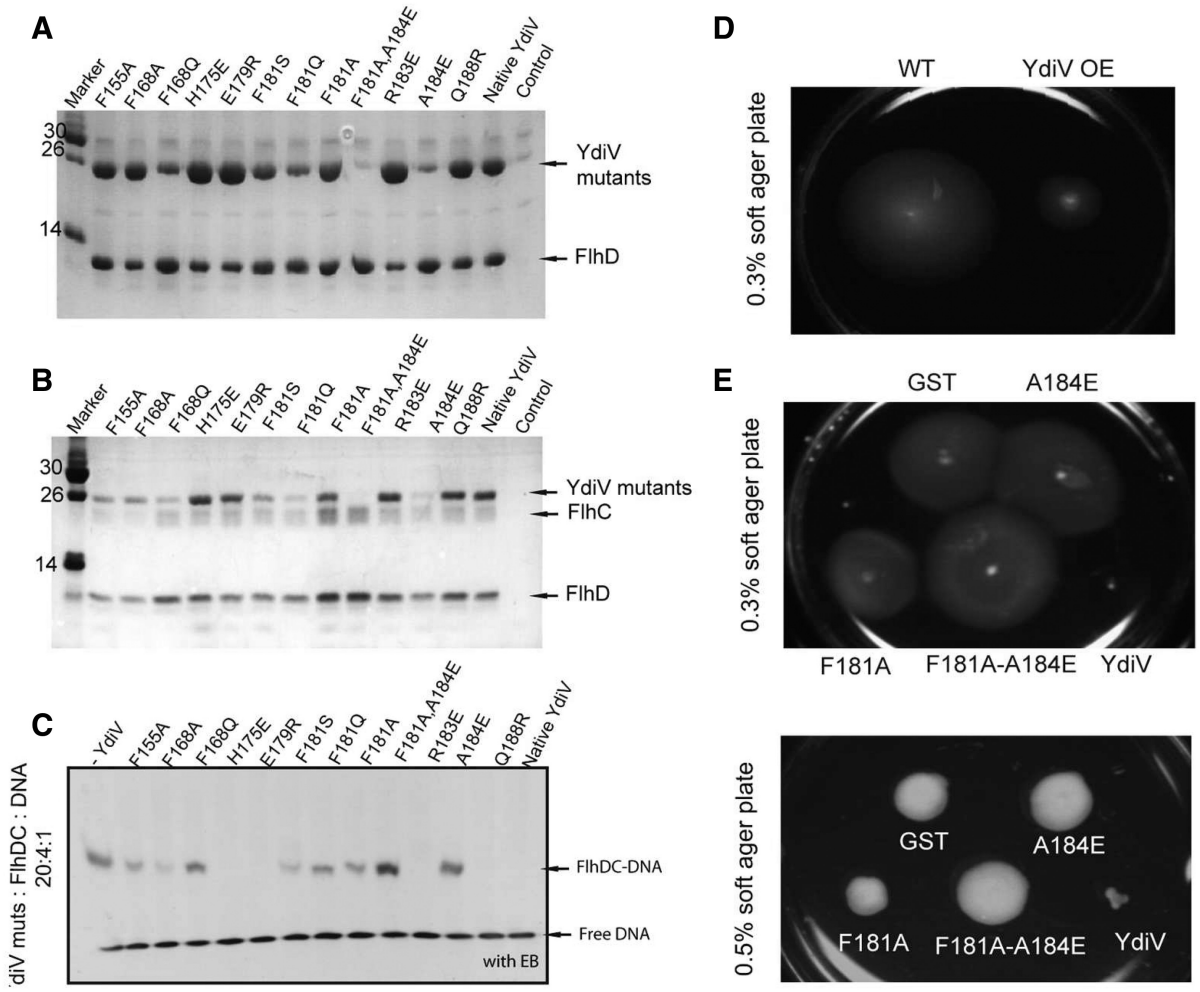
The *in vitro *and *in vivo *functional studies of YdiV mutants. (**A**) Pull down of native/mutants of YdiV by FlhD-His. YdiV did not contain His-tag and was used as the negative control. (**B**) Pull down of the native/mutants of YdiV by FlhD_4_C_2_-His. FlhC contains a C-terminal His-tag, and YdiV was used as the negative control. (**C**) YdiV mutants present different effects to the DNA-binding affinity of FlhD_4_C_2_. Two hundred picomoles of YdiV and its mutants was pre-incubated with 40 pmol FlhD_4_C_2_ for 10 min, and then 10 pmol DNA was added with 10-min incubation before running native gel. The gel was dyed by ethidium bromide. The first lane is a positive control without addition of YdiV. (**D**) The motility of wild-type *E. coli* (WT) and YdiV overproduced (YdiV OE) strain were measured using 0.3% soft agar plate. (**E**) The motilities of five strains with plasmids expressed GST, YdiV and its mutants A184E, F181A, F181A-A184E were measured using soft agar plate containing 100 μg/ml ampicillin and 1 mM IPTG. The upper picture shows results using 0.3% and the lower one using 0.5% soft agar plate.

Residue Ala184 is situated at the center of α8 of YdiV and is next to Asp12 of FlhD in the YdiV–FlhD complex. The A184E mutant completely destroyed the interaction through its oversized side chain and the repulsion to Asp12 of FlhD. This also happened with the double-point mutant F181A–A184E. Another six mutants, F155A, F168A, F168Q, F181S, F181Q and F181A, exhibited reduced affinities to FlhD in varying degrees (Figure 9A).

We also examined the interactions between YdiV mutants and FlhD_4_C_2_. The mutants (F155A, F168A, F168Q, F181S, F181Q, F181A, F181A-A184E and A184E) that are weak in binding FlhD also showed decreased FlhDC-binding affinities in the same degree as with FlhD (Figure 9B). We went on to examine the inhibitory function of YdiV mutants to the DNA-binding activity of FlhD_4_C_2_ with EMSA. The F181A-A184E mutant, which did not bind to FlhD and FlhD_4_C_2_, lost their inhibitory function entirely; the mutants with weak binding affinity to FlhD/FlhD_4_C_2_ revealed decreased inhibitory function. These observations indicate that there is an evidential correlation between the FlhD/FlhD_4_C_2_-binding affinity and the inhibitory function among YdiV mutants (Figure 9C).

We further compared the motility behavior of wild-type *E. coli *and the ones overexpressing YdiV or its mutants on soft agar plates. The size of the zone of swimming by the YdiV overexpressed strain (YdiV OE) was much smaller than wild-type strain, indicating that YdiV inhibits cell motility in *E. coli *(Figure 9D). In comparison, strains that overexpress three mutants (F181A, F181A–A184E and A184E) of YdiV, respectively, all showed similar ability of motility with control strain that harbors GST overexpression (Figure 9E and F).

Our data suggest that the anti-FlhD_4_C_2_ function of YdiV is directly caused by FlhD binding: the YdiV mutants’ lack of FlhD-binding ability also lose all their inhibitory function to FlhD_4_C_2_’s DNA-binding affinity and therefore do not inhibit cell motility *in vivo*; the mutants with decreased binding affinity to FlhD also decrease their inhibitory function to the cell motility in the same degree.

## DISCUSSION

EAL proteins are known to be dimer in solution and catalyze the hydrolysis of c-di-GMP. In this regard, YdiV is an outlier of the family. Sequence analysis shows that 8 out of the 10 conserved catalytic residues are not preserved. Structure analysis indicates that, due to the key residue changes, the potential substrate binding site is no longer compatible for c-di-GMP binding. More importantly, the α8 (176–192) of YdiV differs from typical EAL proteins extensively and abolishes the dimerization activity. Ironically, it is the α8 (176–192) of YdiV that makes the greatest contribution to the interaction with FlhD. Previous bioinformatics studies have showed that 450 out of 1805 EAL-only proteins lack key catalytic residues and do not hydrolyze c-di-GMP (46). Similar to YdiV, those unconventional EAL proteins may not function in c-di-GMP turnover (47).

It has been reported that YdiV can mediate the interaction between the two quorum-sensing systems in *E. coli *in cooperation with its transcription activators SdiA and cAMP (30). This raises a question if there is a crosstalk between quorum-sensing systems and FlhDC transcriptional activity through YdiV (Supplementary Figure S9). Although YdiV cannot bind to c-di-GMP, a large hydrophobic groove is still observed at the potential active site. Electron density map clearly shows the existence of a phosphate and a glycerol molecule in this groove. Thus, small molecules, such as cGMP or cAMP, may bind to YdiV. It is reasonable to expect that binding of ligand may induce significant conformational changes around the active site of YdiV. α6, α7 and α8 of YdiV located around the binding groove could be affected during this process. Consequently, YdiV may lose binding affinity to the FlhD_4_C_2_ complex.

The FlhD_4_C_2_ complex contains four YdiV-binding sites. Two of them are exposed and ready for YdiV binding. The other two sites are buried within the ring-like structure. YdiV squeezes into the ring-like structure to occupy these two sites only when its concentration reaches a certain threshold and saturates the exposed binding sites of FlhD. We have proved that DNA binds the FlhD_4_C_2_ complex through wrapping around FlhC subunits rather than FlhD subunits. Occupation of the two peripheral binding sites by YdiV does not affect the DNA-binding ability of FlhD_4_C_2_ complex.

Our work shows that only when the molar ratio of YdiV:FlhD_4_C_2_ is higher than two, DNA begins to be displaced from the transcriptor complex. The unique mechanism by which YdiV regulates the FlhD_4_C_2_ complex also raises questions if this threshold really exists *in vivo* and if bacteria can benefit from the concentration-dependent mechanism. So far, real-time monitoring of the intracellular concentration of YdiV, FlhD_4_C_2_ and the varieties of related complexes turned out to be very difficult. However, we can expect that, if the YdiV concentration threshold really exists *in vivo*, it would benefit the bacteria. Flagella biogenesis is an energy-consuming and time-consuming process. The switch between the motile and sessile lifestyles is a significant decision for bacteria. In order to prevent unnecessary energy waste, it is better for the bacteria not to switch on or off the corresponding gene transcription too frequently while the external environment is always changing rapidly. Very likely, when the related signal is strong enough and maintains long enough, the intracellular concentration of YdiV slowly accumulates to a certain level for the flagellum biogenesis to begin to decrease. Only when intracellular concentration of YdiV is high enough to saturate all FlhD_4_C_2_ complex, is the biogenesis shut down completely. It appears that the best choice for bacteria is to change their lifestyle only when they have to.

Takaya *et al.* (32) have recently reported that YdiV not only strips FlhD_4_C_2_ from DNA but also facilitates ClpXP protease-mediated FlhD_4_C_2_ degradation. In this regard, when the intracellular concentration of YdiV reaches a threshold, the breakage of the ring-like structure of FlhD_4_C_2_ complex will make YdiV_4_–FlhD_4_C_2_ available to be degraded by ClpXP protease.

**Figure 10.**
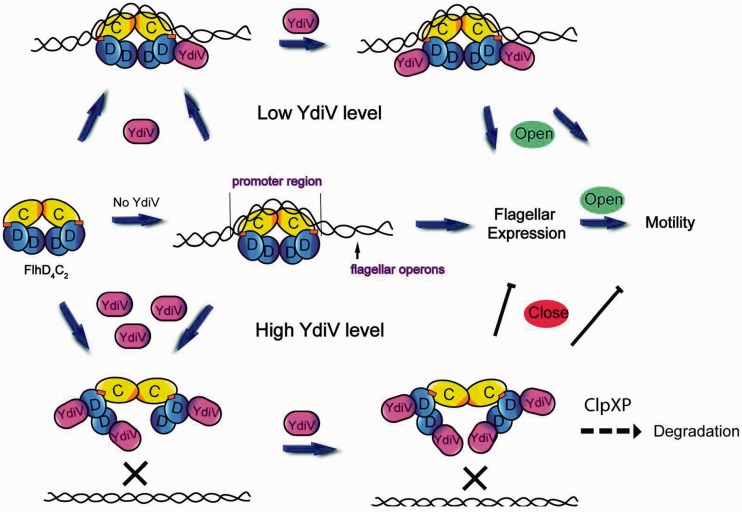
Model for YdiV-mediated motility control. (1) FlhD_4_C_2_ complex recruits DNA through the Zn-cys cluster and the positive-charge-enriched region of FlhC dimer to activate flagellar operons. (2) Through interaction with FlhD, YdiV can compose three-protein complexes with FlhD_4_C_2_. At low concentrations, YdiV only binds to the offside of FlhD_4_C_2_ and do not affect DNA-binding affinity of FlhD_4_C_2_. Further binding of YdiV destroys the ring structure of FlhD_4_C_2_ and demolishes its transcription activator function. As a result, the subsequent expression of flagellar genes is repressed and motility is stopped.

On the basis of our data and previous reports, we propose a model in which YdiV negatively regulates transcriptional activity of FlhD_4_C_2_. In the beginning, FlhD_4_C_2_ binds to the promoter region of flagellar operons and the corresponding genes remain being transcribed. Then the expression of YdiV is triggered by external signal, and YdiV protein starts to bind to the peripheral binding sites of FlhD_4_C_2_. At this stage, gene transcription is not affected. However, if the external signal is strong enough and maintains long enough, the intracellular YdiV concentration eventually reaches a threshold and YdiV begins to squeeze into the ring-like structure of FlhD_4_C_2_ complex. Finally, DNA is displaced from the FlhD_4_C_2_, and FlhD_4_C_2_ is degraded by ClpXP protease. As a result, the subsequent expression of flagellar genes is repressed and motility is stopped (Figure 10).

## ACCESSION NUMBERS

Coordinate and structure factor files of YdiV and YdiV–FlhDC have been deposited in the Protein Data Bank (http://www.rcsb.org/pdb) under ID codes 3TLQ and 4ES4, respectively.

## SUPPLEMENTARY DATA

Supplementary Data are available at NAR Online: Supplementary Table 1, Supplementary Figures 1–9, Supplementary Methods, Supplementary References [33–40].

## FUNDING

Hi-Tech Research and Development Program of China [2006AA02A324]; National Natural Science Foundation of China [31270786 to L.G.]; National Basic Research Program of China [2010CB912401 to H.-W.W.]. Funding for open access charge: National Natural Science Foundation of China [31270786 to L.G.].

*Conflict of interest statement*. None declared.

## Supplementary Material

Supplementary Data

## References

[1] Aldridge P, Hughes KT (2002). Regulation of flagellar assembly. Curr. Opin. Microbiol..

[2] Chevance FF, Hughes KT (2008). Coordinating assembly of a bacterial macromolecular machine. Nat. Rev. Microbiol..

[3] Liu X, Matsumura P (1994). The FlhD/FlhC complex, a transcriptional activator of the Escherichia coli flagellar class II operons. J. Bacteriol..

[4] Pruss BM, Campbell JW, Van Dyk TK, Zhu C, Kogan Y, Matsumura P (2003). FlhD/FlhC is a regulator of anaerobic respiration and the Entner–Doudoroff pathway through induction of the methyl-accepting chemotaxis protein Aer. J. Bacteriol..

[5] Claret L, Hughes C (2000). Functions of the subunits in the FlhD(2)C(2) transcriptional master regulator of bacterial flagellum biogenesis and swarming. J. Mol. Biol..

[6] Claret L, Hughes C (2002). Interaction of the atypical prokaryotic transcription activator FlhD2C2 with early promoters of the flagellar gene hierarchy. J. Mol. Biol..

[7] Wang S, Fleming RT, Westbrook EM, Matsumura P, McKay DB (2006). Structure of the Escherichia coli FlhDC complex, a prokaryotic heteromeric regulator of transcription. J. Mol. Biol..

[8] Soutourina O, Kolb A, Krin E, Laurent-Winter C, Rimsky S, Danchin A, Bertin P (1999). Multiple control of flagellum biosynthesis in Escherichia coli: role of H-NS protein and the cyclic AMP-catabolite activator protein complex in transcription of the flhDC master operon. J. Bacteriol..

[9] Wei BL, Brun-Zinkernagel AM, Simecka JW, Pruss BM, Babitzke P, Romeo T (2001). Positive regulation of motility and flhDC expression by the RNA-binding protein CsrA of Escherichia coli. Mol. Microbiol..

[10] Francez-Charlot A, Laugel B, Van Gemert A, Dubarry N, Wiorowski F, Castanie-Cornet MP, Gutierrez C, Cam K (2003). RcsCDB His-Asp phosphorelay system negatively regulates the flhDC operon in Escherichia coli. Mol. Microbiol..

[11] Tomoyasu T, Takaya A, Isogai E, Yamamoto T (2003). Turnover of FlhD and FlhC, master regulator proteins for Salmonella flagellum biogenesis, by the ATP-dependent ClpXP protease. Mol. Microbiol..

[12] Takaya A, Matsui M, Tomoyasu T, Kaya M, Yamamoto T (2006). The DnaK chaperone machinery converts the native FlhD2C2 hetero-tetramer into a functional transcriptional regulator of flagellar regulon expression in Salmonella. Mol. Microbiol..

[13] Yamamoto S, Kutsukake K (2006). FliT acts as an anti-FlhD2C2 factor in the transcriptional control of the flagellar regulon in Salmonella enterica serovar typhimurium. J. Bacteriol..

[14] Wada T, Morizane T, Abo T, Tominaga A, Inoue-Tanaka K, Kutsukake K (2011). EAL domain protein YdiV acts as an anti-FlhD4C2 factor responsible for nutritional control of the flagellar regulon in Salmonella enterica Serovar Typhimurium. J. Bacteriol..

[15] Imada K, Minamino T, Kinoshita M, Furukawa Y, Namba K (2010). Structural insight into the regulatory mechanisms of interactions of the flagellar type III chaperone FliT with its binding partners. Proc. Natl Acad. Sci. USA.

[16] Schmidt AJ, Ryjenkov DA, Gomelsky M (2005). The ubiquitous protein domain EAL is a cyclic diguanylate-specific phosphodiesterase: enzymatically active and inactive EAL domains. J. Bacteriol..

[17] Tamayo R, Tischler AD, Camilli A (2005). The EAL domain protein VieA is a cyclic diguanylate phosphodiesterase. J. Biol. Chem..

[18] Rao F, Yang Y, Qi Y, Liang ZX (2008). Catalytic mechanism of cyclic di-GMP-specific phosphodiesterase: a study of the EAL domain-containing RocR from Pseudomonas aeruginosa. J. Bacteriol..

[19] Simm R, Morr M, Kader A, Nimtz M, Romling U (2004). GGDEF and EAL domains inversely regulate cyclic di-GMP levels and transition from sessility to motility. Mol. Microbiol..

[20] Hengge R (2009). Principles of c-di-GMP signalling in bacteria. Nat. Rev. Microbiol..

[21] Schirmer T, Jenal U (2009). Structural and mechanistic determinants of c-di-GMP signalling. Nat. Rev. Microbiol..

[22] Romling U (2009). Rationalizing the evolution of EAL domain-based cyclic di-GMP-specific phosphodiesterases. J. Bacteriol..

[23] Tchigvintsev A, Xu X, Singer A, Chang C, Brown G, Proudfoot M, Cui H, Flick R, Anderson WF, Joachimiak A (2010). Structural insight into the mechanism of c-di-GMP hydrolysis by EAL domain phosphodiesterases. J. Mol. Biol..

[24] Hisert KB, MacCoss M, Shiloh MU, Darwin KH, Singh S, Jones RA, Ehrt S, Zhang Z, Gaffney BL, Gandotra S (2005). A glutamate-alanine-leucine (EAL) domain protein of Salmonella controls bacterial survival in mice, antioxidant defence and killing of macrophages: role of cyclic diGMP. Mol. Microbiol..

[25] Simm R, Remminghorst U, Ahmad I, Zakikhany K, Romling U (2009). A role for the EAL-like protein STM1344 in regulation of CsgD expression and motility in Salmonella enterica serovar Typhimurium. J. Bacteriol..

[26] Simms AN, Mobley HL (2008). Multiple genes repress motility in uropathogenic Escherichia coli constitutively expressing type 1 fimbriae. J. Bacteriol..

[27] Wozniak CE, Lee C, Hughes KT (2009). T-POP array identifies EcnR and PefI-SrgD as novel regulators of flagellar gene expression. J. Bacteriol..

[28] Zhao K, Liu M, Burgess RR (2007). Adaptation in bacterial flagellar and motility systems: from regulon members to ‘foraging’-like behavior in E. coli. Nucleic Acids Res..

[29] Wada T, Hatamoto Y, Kutsukake K (2012). Functional and expressional analyses of the anti-FlhD4C2 factor gene ydiV in Escherichia coli. Microbiology.

[30] Zhou X, Meng X, Sun B (2008). An EAL domain protein and cyclic AMP contribute to the interaction between the two quorum sensing systems in Escherichia coli. Cell Res..

[31] Stewart MK, Cummings LA, Johnson ML, Berezow AB, Cookson BT (2011). Regulation of phenotypic heterogeneity permits Salmonella evasion of the host caspase-1 inflammatory response. Proc. Natl Acad. Sci. USA.

[32] Takaya A, Erhardt M, Karata K, Winterberg K, Yamamoto T, Hughes KT (2012). YdiV: a dual function protein that targets FlhDC for ClpXP-dependent degradation by promoting release of DNA-bound FlhDC complex. Mol. Microbiol..

[33] Otwinowski Z, Minor W (1997). Processing of X-ray diffraction data collected in oscillation mode. Method Enzymol..

[34] Terwilliger TC, Berendzen J (1999). Automated MAD and MIR structure solution. Acta Crystallogr. D. Biol. Crystallogr..

[35] Terwilliger TC (2000). Maximum-likelihood density modification. Acta Crystallogr. D Biol. Crystallogr..

[36] Emsley P, Cowtan K (2004). Coot: model-building tools for molecular graphics. Acta Crystallogr. D Biol. Crystallogr..

[37] Adams PD, Grosse-Kunstleve RW, Hung LW, Ioerger TR, McCoy AJ, Moriarty NW, Read RJ, Sacchettini JC, Sauter NK, Terwilliger TC (2002). PHENIX: building new software for automated crystallographic structure determination. Acta Crystallogr. D Biol. Crystallogr..

[38] McCoy AJ, Grosse-Kunstleve RW, Adams PD, Winn MD, Storoni LC, Read RJ (2007). Phaser crystallographic software. J. Appl. Crystallogr..

[39] Tang G, Peng L, Baldwin PR, Mann DS, Jiang W, Rees I, Ludtke SJ (2007). EMAN2: an extensible image processing suite for electron microscopy. J. Struct. Biol..

[40] van Heel M, Harauz G, Orlova EV, Schmidt R, Schatz M (1996). A new generation of the IMAGIC image processing system. J. Struct. Biol..

[41] Barends TR, Hartmann E, Griese JJ, Beitlich T, Kirienko NV, Ryjenkov DA, Reinstein J, Shoeman RL, Gomelsky M, Schlichting I (2009). Structure and mechanism of a bacterial light-regulated cyclic nucleotide phosphodiesterase. Nature.

[42] Minasov G, Padavattan S, Shuvalova L, Brunzelle JS, Miller DJ, Basle A, Massa C, Collart FR, Schirmer T, Anderson WF (2009). Crystal structures of YkuI and its complex with second messenger cyclic Di-GMP suggest catalytic mechanism of phosphodiester bond cleavage by EAL domains. J. Biol. Chem..

[43] Navarro MV, De N, Bae N, Wang Q, Sondermann H (2009). Structural analysis of the GGDEF-EAL domain-containing c-di-GMP receptor FimX. Structure.

[44] Navarro MV, Newell PD, Krasteva PV, Chatterjee D, Madden DR, O'Toole GA, Sondermann H (2011). Structural basis for c-di-GMP-mediated inside-out signaling controlling periplasmic proteolysis. PLoS Biol..

[45] Campos A, Zhang RG, Alkire RW, Matsumura P, Westbrook EM (2001). Crystal structure of the global regulator FlhD from Escherichia coli at 1.8 A resolution. Mol. Microbiol..

[46] Romling U, Amikam D (2006). Cyclic di-GMP as a second messenger. Curr. Opin. Microbiol..

[47] Seshasayee AS, Fraser GM, Luscombe NM (2010). Comparative genomics of cyclic-di-GMP signalling in bacteria: post-translational regulation and catalytic activity. Nucleic Acids Res..

