# Supplementary material for: Structural insight of a concentration-dependent mechanism by which YdiV inhibits Escherichia coli flagellum biogenesis and motility
Source: Nucleic Acids Res. 2012 Sep 21;40(21):11073–85. doi: 10.1093/nar/gks869 (PMC3510510; doi:10.1093/nar/gks869)
Supplement: Supplementary Data [file supp_40_21_11073__index.html]

Structural insight of a concentration-dependent mechanism by which YdiV inhibits Escherichia coli flagellum biogenesis and motility — Structural insight of a concentration-dependent mechanism by which YdiV inhibits Escherichia coli flagellum biogenesis and motility — Supplementary Data 

# Structural insight of a concentration-dependent mechanism by which YdiV inhibits *Escherichia coli* flagellum biogenesis and motility

## Supplementary Data

files

**Files in this Data Supplement:**

- Supplementary Data - pdf file
