# Supplementary material for: Structural insight of a concentration-dependent mechanism by which YdiV inhibits Escherichia coli flagellum biogenesis and motility
Source: Nucleic Acids Res. 2012 Sep 21;40(21):11073–85. doi: 10.1093/nar/gks869 (PMC3510510; doi:10.1093/nar/gks869)
Supplement: Supplementary Data [file supp_gks869_nar-01031-v-2012-File012.pdf]

Supplementary Information

Structural insight of a concentration-dependent mechanism by which YdiV inhibits *Escherichia coli* flagellum biogenesis and motility

Bingqing Li[1], Ning Li[1], Feng Wang[1], Liming Guo[1], Yan Huang[1], Xiuhua Liu[2], Tiandi Wei[1], Deyu Zhu[1], Cuilan Liu[1], Hongfang Pan[1], Sujuan Xu[1], Hong-wei Wang[3], Lichuan Gu[1]*

[1]State Key Laboratory of Microbial Technology, Shandong University, Jinan 250100, China

[2]College of Life Sciences, Hebei University, Baoding 071002, China

[3] Tsinghua-Peking Center for Life Sciences, Center for Structural Biology, School of Life Sciences, Tsinghua University, Beijing 100084, China

*To whom correspondence should be addressed: Tel. 86-531-88362039, E-mail: lcgu@sdu.edu.cn

Supplementary Figures 1-9

Supplementary Table 1

Supplementary Methods

Supplementary Figures 1

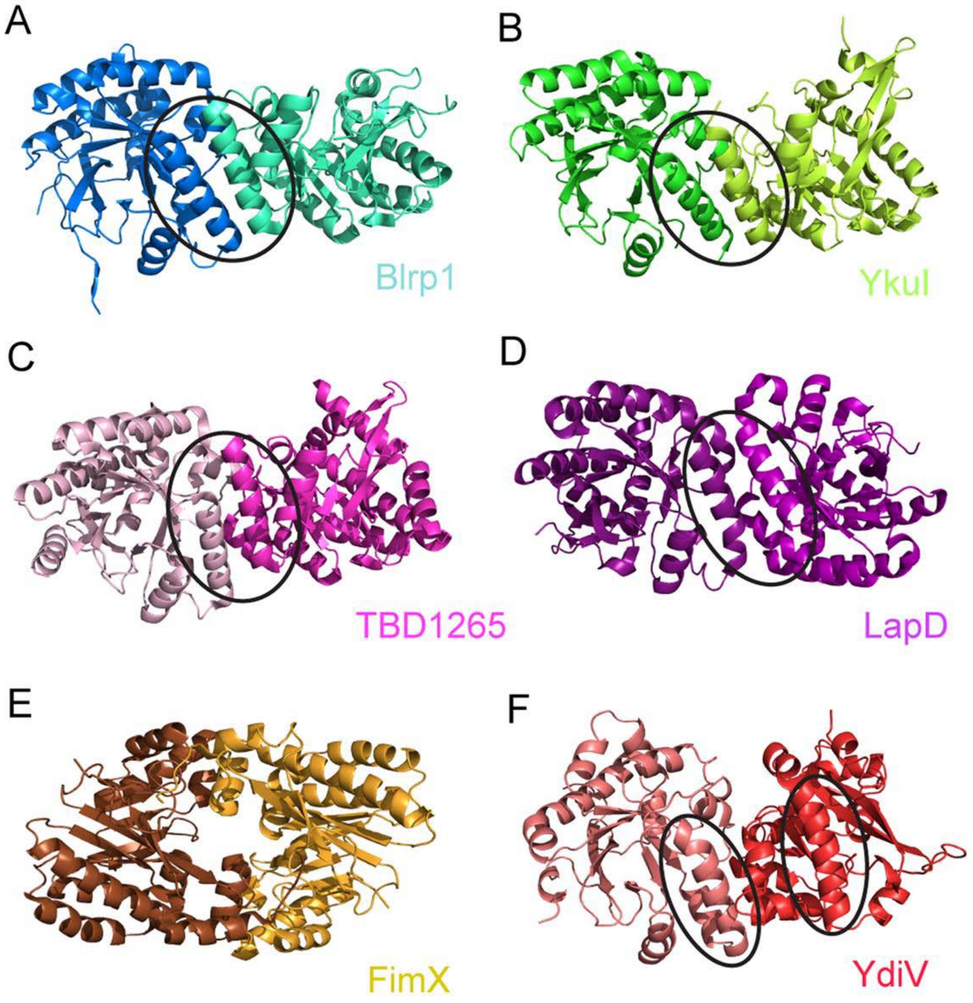

Figure S1. Structural comparion of YdiV with other EAL structures.

All structures are shown in cartoon mode and marked in different colors. The helices which mediate dimerization in Blrp1, YkuI, LapD and TBD1265 structures are highlighted by a black ellipse. Differently, the ones in YdiV structure are far away from each other as shown in F.

Supplementary Figures 2

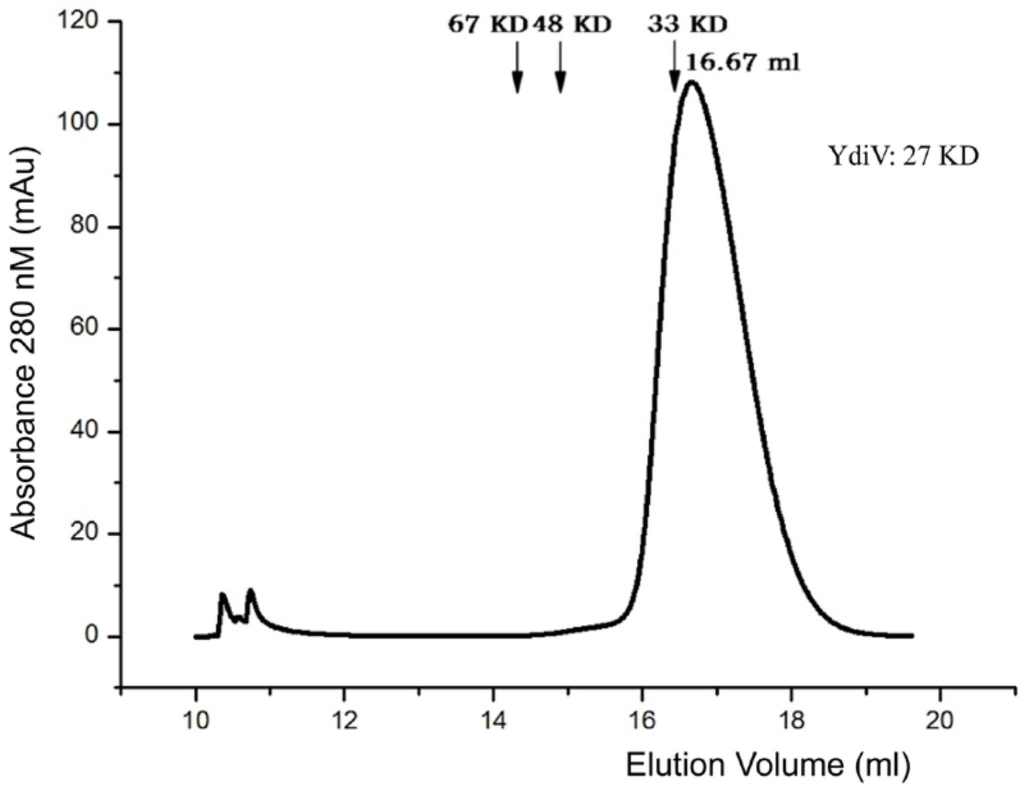

Figure S2. Oligomerization state of YdiV in solution. Standard proteins (arrows) and YdiV were analyzed by size exclusion chromatography. The absorbance (280 nm) peak of YdiV was shown in a curve and the peak value of three standard proteins were labeled in arrows. Three proteins (BSA, VibB and ViuP) were used as standards. BSA has the molecular weight of 67 KDa, and gives the elution volume of 14.1 mL. The molecular weight of VibB-1-215 is 24 KDa. It exists as very stable dimer (48 KDa) in solution and gives the elution volume of 14.8 mL. 33KDa protein ViuP gives the elution volume of 16.2 ml. While YdiV whose molecular weight is 27 KDa eluted at 16.67ml.

Supplementary Figures 3

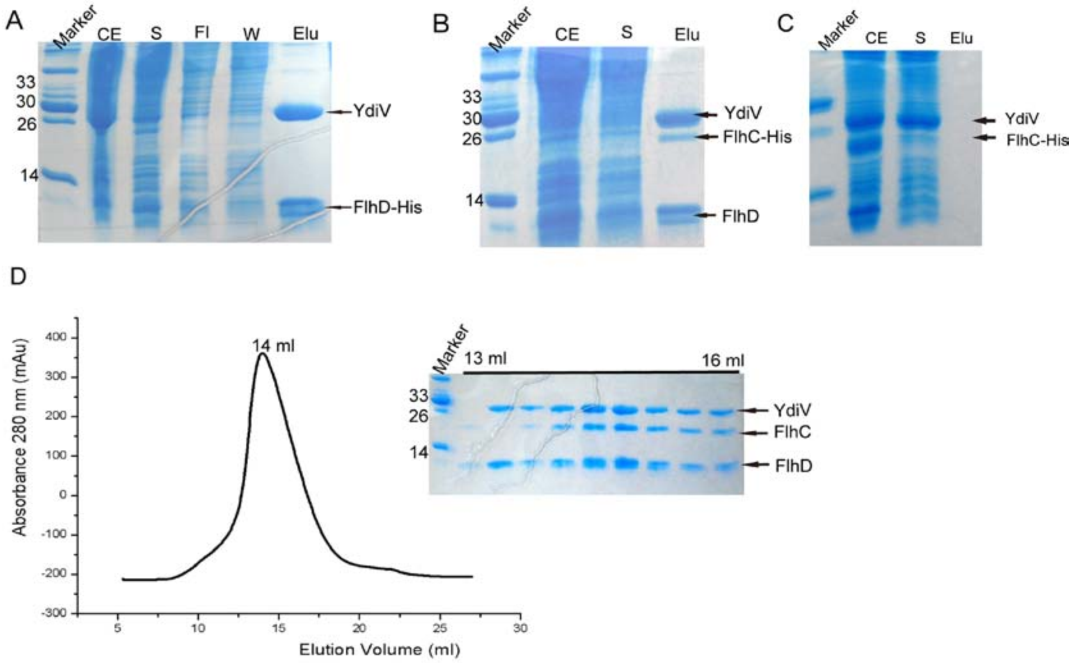

Figure S3. Interaction between *E.coli* YdiV and FlhDC. (A)Co-expression of YdiV and FlhD. YdiV didnot contain his tag while FlhD contained a N-terminal his tag. The two proteins formed a stable dimer. Ce, crude ectract; S, supernatant; Fl, flow through; W, washing; Elu, elution. (B) Co-expression of YdiV and $FlhD_4C_2$. Whole flhDC operon was cloned into pET 21b, by which FlhC contained a C-terminal his tag and YdiV also didnot contain his tag.

(C) Co-expression of YdiV and FlhC. FlhC contained a C-terminal his tag and YdiV also did not contain his tag. (D) Purification of three protein complex (YdiV, FlhD and FlhC) by size exclusion chromatography. SDS-PAGE gel showed that the three proteins coeluted as a stable complex.

Supplementary Figures 4

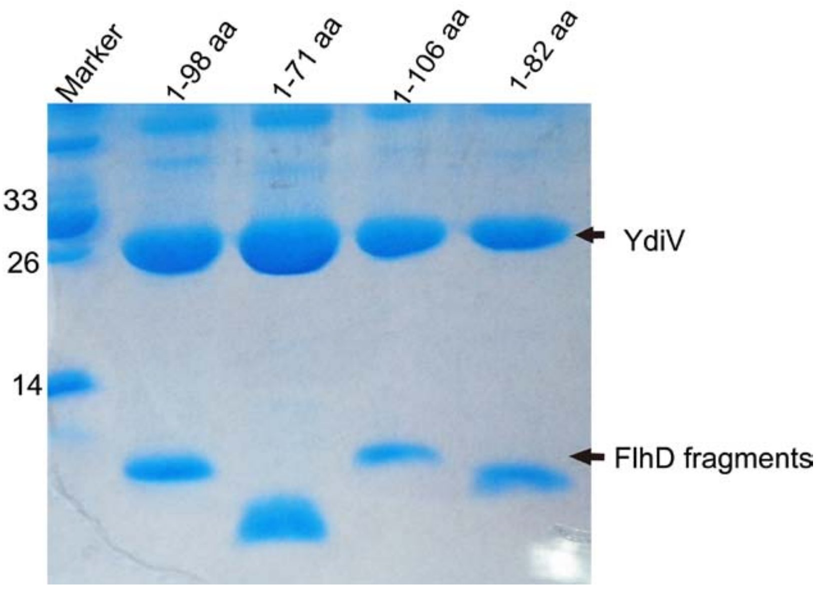

Figure S4. Interaction between four fragments of YdiV and FlhD. Four fragments (1-98aa, 1-77aa, 1-106aa and 1-82aa) were constructed based on the structure of FlhD with a N-terminal his tag. Pull down assay was done as described in Method and Materials.

Supplementary Figures 5

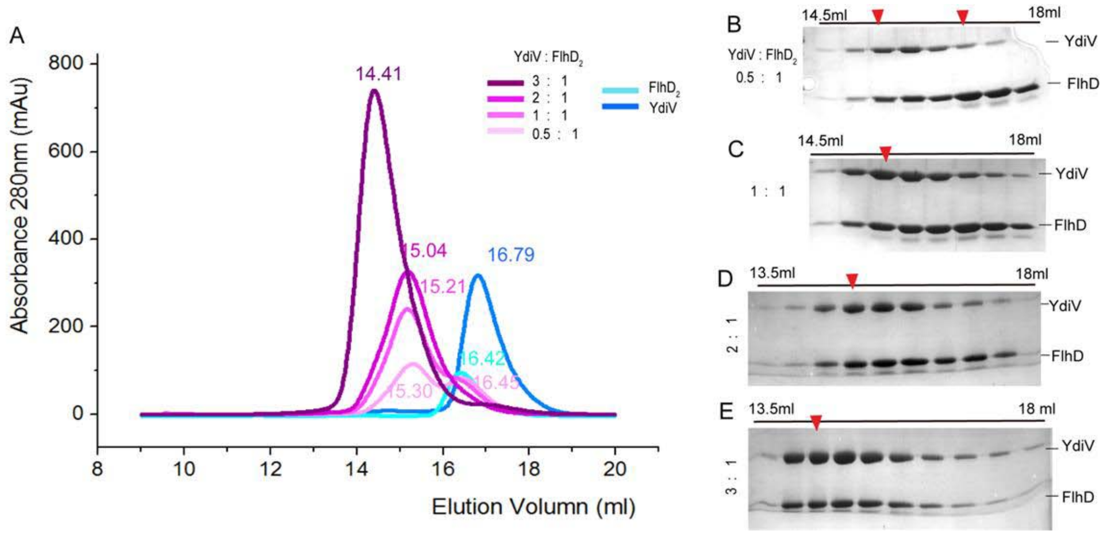

Figure S5. YdiV and FlhD form $YdiV_2$-$FlhD_2$ complex in solution. (A) Size-exclusion chromatography results of 3:1, 2:1, 1:1 or 0.5:1 mixture of YdiV and FlhD dimer. Single YdiV and $FlhD_2$ as controls. The elution volume of every peak value is marked in corresponding colors. (B) SDS-PAGE results of the elutions of 0.5:1mixture from 14.5 ml to 18 ml. The lanes of two peak values are highlighted by red arrow. (C) SDS-PAGE results of the elutions of 1:1mixture from 14.5 ml to 18 ml. Peak value lane is highlighted by red arrow. (D) SDS-PAGE results of the elutions of 2:1mixture from 13.5 ml to 18 ml. Peak value lane is highlighted by red arrow. (E) SDS-PAGE results of the elutions of 3:1mixture from 13.5 ml to 18 ml. Peak value lane is highlighted by red arrow.

Supplementary Figures 6

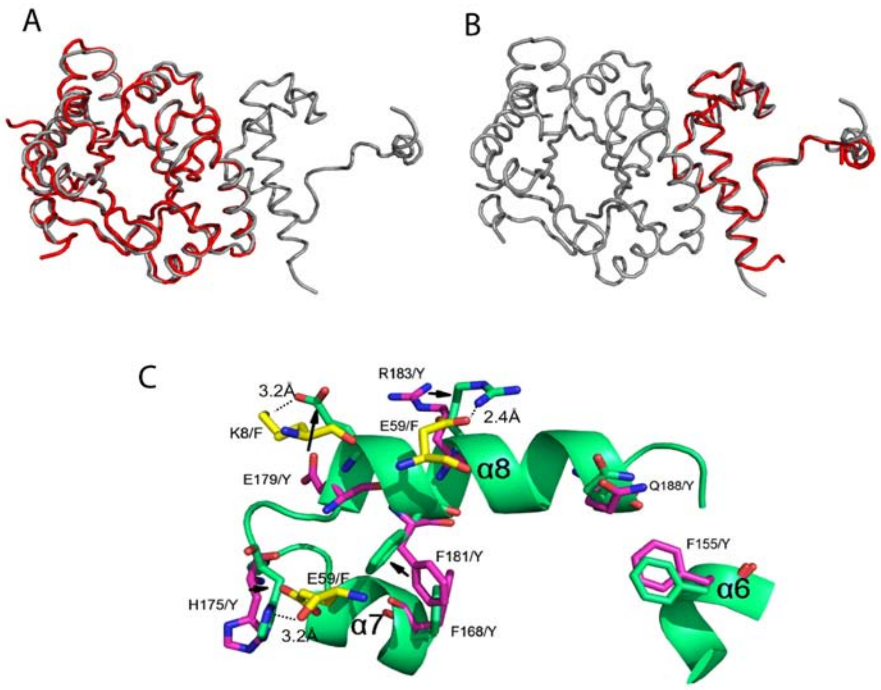

Figure S6. Comparison of YdiV-FlhD complex, YdiV and FlhD structure.

(A) Superposition of YdiV-FlhD and YdiV. Structures of YdiV-FlhD and YdiV are shown in ribbon mode with gray and red color, respectively. (B) Superposition of YdiV-FlhD and FlhD. YdiV-FlhD, gray; FlhD, red. (C) Detail changes of YdiV after binding to FlhD. Residues in YdiV structure are shown in stick mode in pink color while the ones from YdiV-FlhD are shown in green. Residues of FlhD interacting with YdiV are shown in yellow. The positional changes after binding to FlhD are highlighted by arrows. The polar interactions are expressed by dotted line and the distance is labled (Y: from YdiV, F: from FlhD).

Supplementary Figures 7

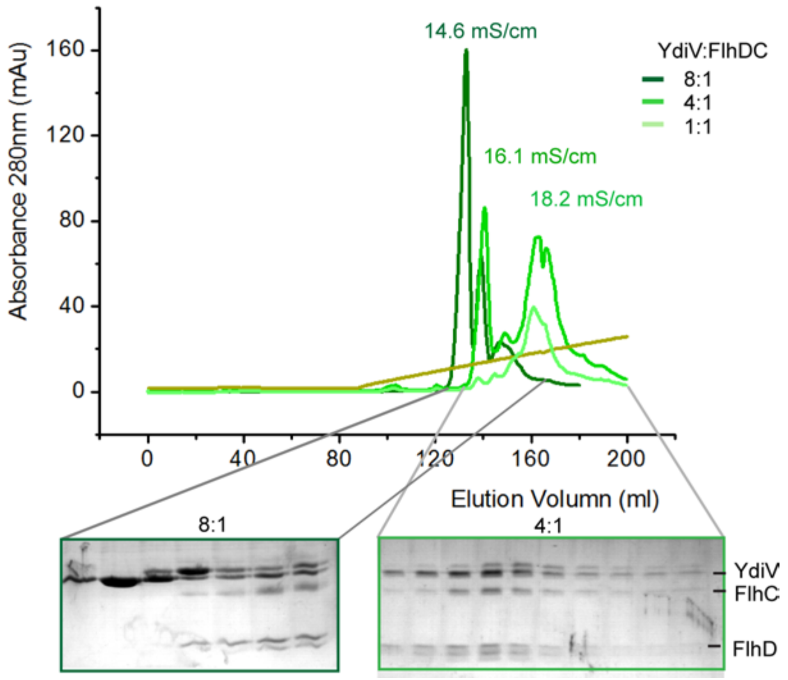

Figure S7. Source Q column analysis of YdiV$_4$-FlhD$_4$C$_2$ complex. The 8:1, 4:1 or 1:1 mixture of YdiV and FlhD$_4$C$_2$ was loaded into Source Q column respectively. Elutions of 8:1, 4:1 mixture were analysed using SDS-PAGE and the results were shown in frames. The conductance of each peak was labeled in corresponding colour.

Supplementary Figures 8

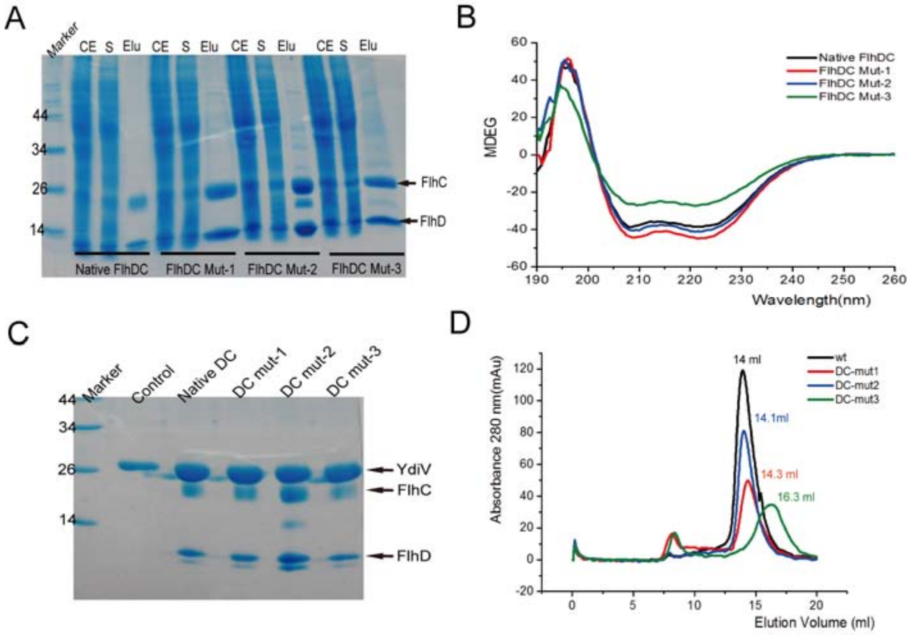

Figure S8. Properties of FlhDC mutants compared with native FlhDC.

(A) Purification results of native FlhDC and FlhDC mutants after nickel column. Ce, crude ectract; S, supernatant; Elu, elution. (B) Circular dichromism results of native FlhDC and FlhDC mutants. All of the proteins showed very similar CD spectre, indicating that little conformational changes before and after mutation. (C) Pull-down results between YdiV and FlhDC mutants. The whole flhDC operon was cloned into pET21b in which FlhC contained a C-terminal His-tag while YdiV without his tag. (D) Size-exclusion chromatography analysis of native FlhDC and FlhDC mutants.

Supplementary Figures 9

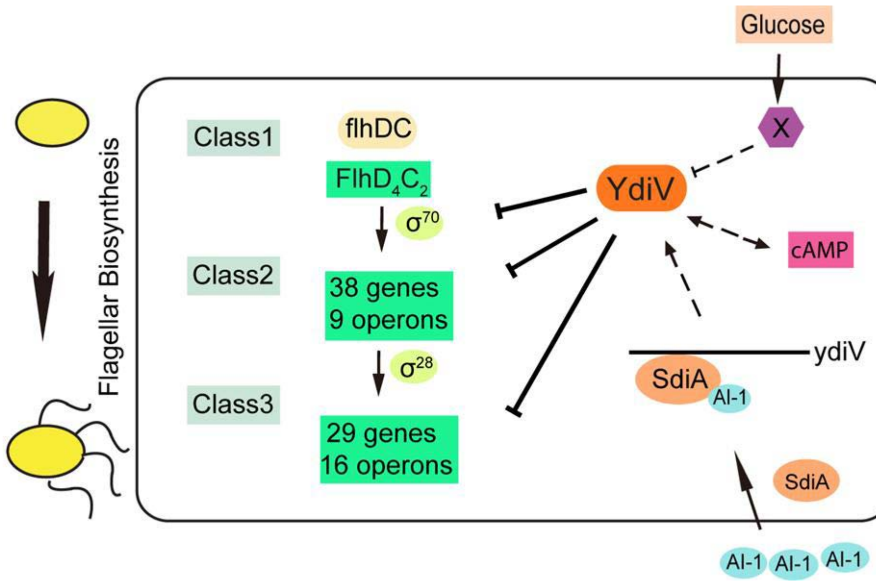

Figure S9. Model of YdiV pathway. Extracellular signals (glucose and autoinducer-1) inhibit or activate YdiV through some unknown mechanism and then turn on or turn off flagellar biosynthesis. In this regard, motility of bacteria can be regulated by quorum sensing signals or nutrition signals receipting by YdiV and acting through the interaction between YdiV and $FlhD_4C_2$.

Supplementary Table 1

Table S1. Data collection and refinement statistics

	YdiV	YdiV-FlhD
**Data collection**		
Space group	P21	P3121
Cell dimensions		
a, *b*, *c* (Å)	49.58, 50.35, 111.07	132.49,132.49,145.69
α, β, γ (°)	90, 98.62, 90	90, 90, 120
Resolution (Å)	50-1.91 (1.98-1.91)*	50-2.90 (3.00-2.90)
R_{sym} or R_{merge}	0.081 (0.197)	0.095 (0.748)
$I / \sigma I$	17.53 (5.48)	46.68 (5.03)
Completeness (%)	98.8 (94.1)	99.8 (100)
Redundancy	3.5 (3.0)	10.7 (11.0)
**Refinement**		
Resolution (Å)	39.635-1.909	39.165-2.897
No. reflections	40814	31417
R_{work} / R_{free}	0.172/0.2	0.245/0.283
No. atoms		
Protein	3797	9080
PO4	30	-
CRY	18	-
Water	391	0
B-factors		
Protein	14.785	82.564
PO4	25.246	-
CRY	29.152	-
Water	23.56	-
R.m.s. deviations		

Bond lengths (Å)	0.007	0.011
Bond angles (°)	1.076	1.367
Ramachandran plot (%)		
Most favored (%)	92.9	89.7
Additionally allowed (%)	7.1	10.3
Generously allowed (%)	0.5	0.8
Disallowed (%)	0	0

*Highest resolution shell is shown in parenthesis. One crystal was used for each dataset.

Supplementary Methods

Protein expression and purification

The ydiV and flhD genes were PCR amplified from *Escherichia coli* str. K-12 substr. MG1655 genome and subcloned into pET29b and pGL01, a modified vector based on pET15b with a PPase cleavage site to remove 6×His. YdiV was expressed in *E. coli* BL21(DE3) using LB medium with 100 μg/ml Ampicillin. When OD600 reached 0.6, cultures were cooled to 15°C and induced overnight by 0.1 mM IPTG. Harvested cells were resuspended in lysis buffer (25 mM Tris-HCl , pH 8.0, 200 mM NaCl) and lysed by sonication. After centrifugation at 28,500×g for 45min, YdiV was purified by Ni^{2+}-NTA affinity column, ion exchange column Source Q and Superdex 200 successively.

Two isoleucines (Ile43 and Ile182) was replaced by methionines to increase signal for SAD. Se-Met-YdiV was expressed in *E. coli* BL21(DE3) using M9 medium. L-Seleno-methionnine was added to the culture when OD600 reached 0.5. The purification procedure of Se-Met-YdiV was the same with native YdiV.

YdiV-FlhD complex was obtained by co-expression in *E. coli* BL21 (DE3) with the same expression condition of YdiV. After Ni^{2+}-NTA affinity column, YdiV-FlhD complex was lysed by 0.25 mg/ml trypsin for 30 min and purified by ion exchange column Source Q and Superdex 200 chromatography immediately.

Whole flhDC operon was cloned into pET21b in which FlhC contained a C-terminal his tag. $FlhD_4C_2$ complex were obtained by co-expression and further purified by Ni^{2+}-NTA affinity column, ion exchange column Source Q and Superdex 200.

Site-directed mutagenesis

Mutants of YdiV (F155A, F168A, F168Q, H175E, E179R, F181S, F181Q, F181A, F181A-A184E, R183E, A184E and Q188R) and FlhDC (DC-Mut-1, DC-Mut-2 and DC-Mut-3) were constructed using the two-step PCR strategy and cloned into pGL01,

respectively. Three YdiV mutants (A184E, F181A and F181A-A184E) were also cloned into pGEX-6P-1 and transformed into *Escherichia coli* str. K-12 substr. MG1655 for further motility studies. All proteins were expressed and purified using the method as described above. Purified proteins were stored at -80°C.

Crystallization and Structure Determination

YdiV were concentrated to 9 mg/ml. Crystals were grown using hanging drop vapour diffusion at 20°C. The crystallization buffer contains 2.0 M Sodium/Potassium Phosphate pH 5.2. The SeMet crystals were obtained in the condition contains 0.2 M $(NH_4)_2SO_4$, 0.1 M MES monohydrate pH 6.5 and 18% Polyethylene glycol monomethyl ether 5,000. Both native and anomalous diffraction data were collected at Shanghai Synchrotron Radiation facility (SSRF) beamline BL17u1. The crystals of YdiV alone belong to space group P21 and contain two molecules per asymmetric unit. The unit cell dimensions are a = 49.58 Å, b = 50.35 Å, c = 111.07Å, andβ = 98.62. The crystal of YdiV -FlhD belongs to space group P3121 and contains eight molecules per asymmetric unit. The unit cell dimensions are a = 132.49Å, b = 132.49Å, c = 145.69Å.To prevent radiation damage, crystals were equilibrated in a cryoproctetant buffer containing 15% glycerol (v/v) plus reservoir buffer and then flash frozen in a 100K nitrogen stream. The data sets were processed using the HKL2000 software suite (33). Structure of YdiV alone was solved by single anomalous dispersion (SAD) phasing. Five Se sites were found using the program SOLVE (34). Initial single anomalous dispersion phases were then improved and the chain was automatically traced using the program RESOLVE (35). The atomic model was built using COOT (36) and refined using PHENIX (37). Data collection and structure refinement statistics are summarized in Table 1. The structure of YdiV-FlhD was determined at 2.9Å resolution, with the molecular replacement approach using PHASER (38) with the FlhD structure (PDB code: 1G8E) and our YdiV structure as searching models. The final model contains four YdiV and four FlhD molecules in the asymmetric unit. Structural figures were generated using PyMol (http://www.pymol.org).

Size-exclusion chromatography

Purified proteins (about 0.4 mg) were injected to size exclusion chromatography using a superdex 200 column equilibrated by a buffer contains 10 mM Tris-HCl (pH 8.0) and 100 mM NaCl. Three proteins (BSA, VibB and ViuP) were used as standards. For complex study, two proteins (YdiV and $FlhD_4C_2$) were mixed in different mixing ratio (5:1~1:1) for 10 min at room temperature and injected to size exclusion chromatography using a superdex 200 column. All data were processed by Origin.

Protein Pull-down Assay

Bait protein (with his tag) were prepared as described above. His-tag of prey protein were removed by PPase during purification. Approximately 0.05 mg of bait protein was immobilized onto Ni^{2+}-NTA beads and then incubated with 0.3 mg of prey protein at 4°C for 30 min. The mixture was washed three times using buffer containing 25 mM Tris-HCl (pH8.0), 100 mM NaCl. Protein were eluted with elution buffer containing 25 mM Tris-HCl (pH8.0), 100 mM NaCl, 250 mM imidazole. Then the elution samples were analyzed by SDS-PAGE with coomassie blue staining. 0.2-0.4 mg of prey protein were incubated with Ni^{2+}-NTA beads alone as a negative control.

EMSA Experiment

A 49 bp DNA fragment containing the $FlhD_4C_2$ box of the flhB promoter was synthesized as target DNA. 10 pmol DNA was pre-incubated with different radios proteins in a reaction buffer contains 20 mM Tris-HCl (pH8.0), 100 mM NaCl,1 mM $MgCl_2$, 1 mM $ZnCl_2$ and 4%(v/v) glycerol for 10 minutes. Then samples were analysed using a native 5% polyacrylamide gel at 4 ^{o}C in 0.5TBE buffer (46 mM Tris base, 46 mM boric acid, 1 mM EDTA) for 1 h and dyed by EB for 10 min. Some gels were also dyed by coomassie brilliant blue.

Swarming Motility Assay

Motility was evaluated using 0.3% or 0.5% soft agar plates as described before. Briefly, single colonies were poked into the plates using toothpicks and incubated for 6 h at 37°C. For motility

assays requiring the induction of pGEX construct, colonies were picked from plates that contained 1 mM IPTG. At least six independent colonies were checked for each strain.

Negative stain EM sample preparation and single particle image analysis

The gel-filtration purified FlhDC-YdiV complex were diluted to 50-80 nM in the elution buffer and immediately applied to glow-discharged holey carbon grids with a thin layer of carbon over the holes. After 1 min, the samples were stained consecutively in 3 droplets of 2% (w/v) uranyl acetate solution and the remaining stain was removed by gentle blotting with filter paper. The samples were examined using an FEI F20 electron microscope equipped with a field emission gun operated at 200 kV acceleration voltage using a nominal magnification of 50,000. Images were recorded on a 4k x 4k Ultrascan4000 CCD camera (Gatan) using low-dose mode with an exposure dose of 20–30 e/$Å^2$. The defocus used to collect the raw image was -1.2 μm to -1.5 μm. The electron micrographs had a pixel size of 2.2Å and were directly used for image processing. We used EMAN2 package to perform semi-automatic particle picking and to box the particles from the raw micrographs into boxes of 80 x 80 square pixels for the samples (39). The particles were normalized and high- and low-pass filtered prior all image processing procedures. About 30,000 raw particles of FlhDC-YdiV complexes were collected to two-dimensional reference-free alignment and classification using multivariate statistical analysis and multireference alignment in IMAGIC-4D (40) to a total of 200 classes.
